# Antifungal Attributes of *Lactobacillus plantarum* MYS6 against Fumonisin Producing *Fusarium proliferatum* Associated with Poultry Feeds

**DOI:** 10.1371/journal.pone.0155122

**Published:** 2016-06-10

**Authors:** B. V. Deepthi, K. Poornachandra Rao, G. Chennapa, M. K. Naik, K. T. Chandrashekara, M. Y. Sreenivasa

**Affiliations:** 1 Department of Studies in Microbiology, University of Mysore, Mysuru, Karnataka, India; 2 Department of Plant Pathology, College of Agriculture Science, UAS, Raichur, Karnataka, India; 3 Institution of Excellence, Vijnana Bhavan, University of Mysore, Mysuru, Karnataka, India; The University of Wisconsin—Madison, UNITED STATES

## Abstract

Fumonisins, being common in occurrence in maize-based feeds, pose a great threat to animal and human health. The present study is aimed at determining the antifungal activity of *Lactobacillus plantarum* MYS6 against a fumonisin producing fungus, *Fusarium proliferatum* MYS9. The isolate was subjected to standard tests for determining its probiotic attributes and antifungal properties. *L*. *plantarum* MYS6 thrived well at pH 3.0 and 6.0, and exhibited strong resistance up to 3% bile. The isolate showed a high degree of cell surface hydrophobicity corresponding to its strong adhesion to chicken crop epithelial cells. Co-inoculation with the fungus on modified de Man Rogosa Sharpe medium revealed the inhibitory effect of *L*. *plantarum* MYS6 on fungal growth and biomass. Observation using scanning electron microscopy showed distortion of hyphal structures, swollen tips and disrupted conidia. Conidia germination inhibition assay restrained germination and showed deformed hyphae. The bioprotective feature of the isolate was evident by the inhibition of fungal development in maize-kernel treated with the cell free supernatant of *L*. *plantarum* MYS6. Both the isolate and its extracellular metabolites lowered fumonisin content in feed model up to 0.505 mg/Kg of feed and 0.3125 mg/Kg of feed respectively when compared to the level of 0.870 mg/Kg of feed in control. The major antifungal compounds produced by the isolate were 10-Octadecenoic acid, methyl ester; palmitic acid, methyl ester; heptadecanoic acid, 16-methyl ester; stearic acid and lauric acid. *L*. *plantarum* MYS6 reduced 61.7% of fumonisin possibly by a binding mechanism. These findings suggest the application of *L*. *plantarum* MYS6 as an efficient probiotic additive and biocontrol agent in feed used in poultry industry. Additionally, the antifungal metabolites pose a conspicuous inhibition of *Fusarium* growth and fumonisin production.

## 1. Introduction

Deterioration of food/feed stuffs due to fungal colonization and concomitant production of mycotoxins is a serious problem, especially in the wake of fungi acquiring resistance to many commonly used chemical preservatives. Fungal spoilage may occur during pre-harvest, harvest or post-harvest stages due to non-scientific agricultural practices, poor storage facilities and unfavorable environmental conditions. In addition to the food losses due to fungal growth, their mycotoxins lead to serious health hazards in human and animals. *Fusarium proliferatum* is a food contaminant known to colonize and produce fumonisin which is a carcinogenic agent [1]. It is a common contaminant of maize and maize based products worldwide. Considerable interest in fumonisin emerged after discovering its high toxicity responsible for animal diseases like leukoencephalomalacia, porcine pulmonary edema, etc. [2]. Moreover, fumonisins have been associated with nephrotoxic, hepatotoxic and immunosuppressing effects in various animals including poultry and rats [3]. On account of the structural analogous nature of fumonisins, particulary FB1 to ceramide synthase, it inhibits sphingolipid metabolism and interferes with cell regulation [4].

Detoxification of toxins cannot be fully achieved as their production is modulated by environmental factors. Although physical and chemical methods have been used [5], they are not very effective or difficult to incorporate into the production process [6]. Moreover, fungi have acquired resistance to many of the conventional chemical treatments [7]. Therefore, an effective alternative strategy would be the use of microorganisms which can control fungal growth and thus overcome the production of mycotoxins. Among these, lactic acid bacteria (LAB) are of considerable interest on account of their detoxifying capacity, probiotic potential and ability to produce an array of antimicrobial metabolites. The mechanism by which LAB detoxifies mycotoxin remains to be elucidated; however, several reports suggest the binding nature of LAB to the mycotoxin moieties. Niderkorn et al. [8] showed the binding ability of *Lactobacillus*, *Leuconostoc*, *Enterococcus*, *Pediococcus*, *Propionibacterium*, and *Streptococcus* and explained that tricarballylic acid chains of fumonisin molecules and peptidoglycan of LAB could be involved in the binding process. Both viable and non-viable LAB could bind fumonisin *in vitro* based on pH, genus, bacterial density and fumonisin analogue (FB2 > FB1) [9]. Binding of other major mycotoxins such as aflatoxin B1, zearalenone [10, 11] and some trichothecenes [12] by some probiotic LAB have also been reported *in vitro*. Among LAB, different strains of *Lactobacillus* isolated from fermented food products such as sourdough, grass silage, vegetable products [13, 14, 15] etc. have been shown to possess antifungal activity. Lactobacilli also produce antifungal metabolites such as organic acids, hydrogen peroxide, proteinaceous compounds, hydroxyl fatty acids and phenolic compounds [14, 16]. Also bacteriocin-like substances and other low and medium molecular weight compounds produced by LAB have shown antifungal property [17].

The present study aimed at evaluating the antifungal activity of a LAB against fumonisin producing *Fusarium proliferatum* occurring on poultry feeds. Our study describes the isolation, identification and assessment of probiotic attributes of the LAB from a traditional fermented food. The inhibitory effect of the isolate against *F*. *proliferatum* and its production of fumonisin were determined by employing various antifungal assays. The study also reports the extraction and purification of antifungal metabolites obtained from the isolate by GC/MS. Furthermore, alterations in hyphal morphology and conidia exposed to LAB and its supernatant were observed by SEM. In addition, we also made an attempt to know the possible mechanism involved in the detoxification of fumonisins by LAB.

## 2. Material and Methods

### 2.1 Isolation, identification and preparation of spore suspension of *Fusarium*

*Fusarium proliferatum* was isolated from poultry feed mixture collected from poultry farm, Mysuru, Karnataka, India by serial dilution spread plate method on potato dextrose agar (PDA). The pure culture of the fungus was obtained by single spore isolation and grown on Czapek Dox Agar (CZA) slants under 12:12 h light-dark conditions at 28°C ± 2°C for 5 days and maintained at 4°C for further studies.

Genomic DNA was extracted from *F*. *proliferatum* by the conventional phenol-chloroform method [18]. The isolate was subjected to polymerase chain reaction (PCR) screening for species specific primers, Fp3-F (5’CGGCCACCAGAGGATGTG3’) and Fp-4R (5’CAACACGAATCGCTTCCTGAC3’) and for *fum*1 gene, which encodes fumonisin using the primer pairs rp32 (5’ACAAGTGTCCTTGGGGTCCAGG3’) and rp33 (5’GATGCTCTTGGAAGTGGCCTACG3’) [19, 20]. Amplicons were sequenced and confirmed by BLAST analysis. Fumonisin producing ability of the species was further analyzed by LC/MS (Waters Acquity/Synapt G2, USA).

*F*. *proliferatum* spore suspension was prepared by culturing on PDA slants and incubating at 28°C ± 2°C for 5–7 days. After incubation, spores were harvested by adding 0.1% Tween 80 followed by gentle shaking. Spore concentration was adjusted using hemocytometer to 10^6^ spores/mL.

### 2.2. Isolation, identification and characterization of LAB

LAB was isolated from fermented pomegranate wine collected from Coorg, Karnataka. The sample was serially diluted and spread on de Man Rogosa Sharpe (MRS) agar plates and incubated at 37°C for 48 h under anaerobic condition. The visible, discrete colonies were sub-cultured on to MRS broth. The LAB was characterized on the basis of morphological, biochemical and physiological parameters [21].

#### 2.2.1. Screening of LAB for antifungal activity

Antifungal activity of the isolated LAB against *Fusarium proliferatum* was evaluated by agar overlay and agar well diffusion methods as described by Magnusson and Schnurer [22] with minor modifications. The agar overlay assay was performed using MRS agar plates on which LAB were streaked as two lines and incubated anaerobically at 37°C for 48 h. Then 100μL spore suspension (10^6^ spores/mL) of *F*. *proliferatum* MYS9 (Fp MYS9) were evenly mixed with malt extract soft agar (2% malt extract, 0.7% agar) and overlaid on the incubated MRS agar plates. The plates were then incubated aerobically at 28°C ± 2°C for 4 days. The plates were examined for clear inhibitory zones around the streaked area of the colonies (- no visible inhibition; + visible inhibition only above the LAB streak; ++ inhibition area of 3–8% plate area; +++ inhibition area >8% plate area). For agar well diffusion assay, PDA plates were prepared and spread with 100μL spore suspension of Fp MYS9 (10^6^spores/mL). Wells of 7.5mm diameter were prepared on PDA plates and were dispensed with 100μL, 250μL and 500μL of cell free supernatant (CFS) of each LAB isolate. The plates were incubated at 28°C ± 2°C for 72 h and examined for inhibitory zone. The LAB isolates showing potent antifungal activity were selected for further studies. Experiments were performed in triplicates.

#### 2.2.2. Species identification of LAB

The species of LAB was identified by partial sequencing of 16S rDNA by the primer pairs 27F (5’AGAGTTTGATCCTGGCTCAG3’) [23] and 519R (5’GWATTACCGCGGCKGCTG3’) [24]. The amplicon of 16S rDNA was sequenced and analyzed. The phylogenetic tree was constructed using MEGA 5.1 software by neighbor joining algorithm. The nucleotide sequence was submitted to NCBI Genbank.

#### 2.2.3. Assessment of probiotic attributes

Tolerance of *Lactobacillus plantarum* MYS6 (Lp MYS6) to acidic pH was examined as described by Salah et al. [25] with minor modifications. Lp MYS6 (10^6^ CFU/mL) was inoculated into phosphate-buffered saline (PBS) of different pH (2.0, 3.0 and 6.0), mixed thoroughly, and incubated for 0, 1 and 3 h. After incubation, cell survivability was determined by serial dilution and plating on MRS agar. Growth of LpMYS6 in the presence of Ox gall (2 and 3%) was evaluated as described by Ehrmann et al. [26]. Susceptibility of Lp MYS6 to a panel of 9 antibiotics was determined according to Kirby Bauer disc diffusion method following CLSI guidelines [27]. The haemolytic activity of the isolate was examined as per the procedure of Maragkoudakis et al. [28] and observed for α-haemolysis, β-haemolysis and no-haemolysis around the colonies.

The cell surface hydrophobicity assay was conducted by microbial adhesion to hydrocarbons (MATH) as described by Lee et al. [29] with slight modifications. The concentration of LpMYS6 was adjusted to 10^6^ CFU/mL in 50 mM PBS. One mL of the bacterial suspension (A_0_) was mixed with 1.0 mL each of n-Hexadecane and xylene and vortexed for 2 min. The mixture was allowed to separate at 37°C for 30 min. The absorbance of the upper aqueous layer (A) was measured at 600 nm. Percent hydrophobicity was calculated as follows: [(A_0_ –A)/A_0_] ×100. *Lactobacillus plantarum* MTCC 9483 was used as the reference strain in the present investigation.

#### 2.2.4. *In vitro* adhesion to chicken crop epithelial cells

The adhesion capacity of LpMYS6 to chicken crop epithelial cells was determined *in vitro* according to Jakava-Viljanen and Palva [30]. The chicken crop was maintained in PBS at 4°C for 30 min to remove surface mucus and washed thrice with potassium phosphate buffer (pH 7.4). Epithelial cells of the chicken crop tissue were gently scrapped using a sterile cover slip and the scrapings were suspended in PBS. The epithelial cells were microscopically examined to ensure the elimination of adhering commensal bacteria and cells were then diluted to approximately 5 × 10^6^ cells/mL. One hundred μL of LpMYS6 (10^6^ CFU/mL) in 400 μL of epithelial cells was prepared and mixed well followed by incubating in a water bath at 37°C for 30 min. After incubation, the mixture was centrifuged at 3000 rpm for 3 min and the pellet was washed twice with sterile PBS to remove unattached bacteria. It was then resuspended in 100 μL of PBS. The preparation was then stained with Acridine Orange and observed under Fluorescent Microscope (Carl Zeiss, Germany). Bacterial adhesion was examined in 10 microscopic fields and scored positive if a minimum of 10 bacteria were found adhering to each epithelial cell.

### 2.3. Antifungal activity assays

The following antifungal assays were performed by using either the cell suspension or supernatant of Lp MYS6. To prepare cell free supernatant, 18h old culture was centrifuged at 10000 rpm for 12 min and filtered sterilized using Whatman No. 1 filter disc.

#### 2.3.1. Co-inoculation assay

A modified MRS medium was designed which allowed the growth and survival of both *Lactobacillus* and *Fusarium*. Erlenmeyer flasks with 50 mL of modified MRS (Bacteriological Peptone 5 g/L, mycological peptone 5 g/L, beef extract 10 g/L, yeast extract 5 g/L, dextrose 20 g/L, MgSO_4_ 0.10 g/L, MnSO_4_ 0.05 g/L, K_2_HPO_4_2 g/L) medium devoid of antifungal substances such as polysorbate 80, ammonium citrate and sodium acetate were inoculated with 100μL of LpMYS6 (10^6^ CFU/mL) and Fp MYS9 (10^6^ spores/mL). Cultures were incubated at 30°C for 3, 7, 10 and 14 days. After incubation, mycelial biomass was weighed and log CFU mL^-1^ of Lp MYS6 was also determined at respective intervals. The control consisted of 100 μL Fp MYS9 (10^6^ spores/mL) in 50 mL modified MRS medium. The assay was performed in triplicate.

#### 2.3.2. *Fusarium* biomass inhibition

Fungal biomass inhibition was carried out by using the cell free supernatant of LpMYS6 as per the protocol [31]. Different concentrations (2, 4, 6, 8 and 10%) of cell-free supernatant of Lp MYS6 (CFS-Lp MYS6) were prepared in 50 mL potato dextrose medium and inoculated with a 7.5 mm diameter fungal disc. The flasks were incubated at 28°C ± 2°C for 10 days. Flask without CFS served as control. After incubation, fungal mat was harvested, filtered (Whatman No. 1), and dried in hot air oven at 50°C for two hours. Fungal biomass of each treatment was weighed and compared with the control. The experiment was conducted in triplicate.

#### 2.3.3. Scanning electron microscopy (SEM) analysis

SEM was employed to visualize the hyphal morphology of Fp MYS9 and to know the possible mechanism between Lp MYS6 and Fp MYS9. For SEM analysis, modified MRS plates were prepared and wells of 7.5 mm diameter were made. Wells were dispensed with a mixture of 100 μL of LpMYS6 (10^6^ CFU/mL) and 100 μL of Fp MYS9 (10^6^ spores/mL). Another set of reaction consisted of 100 μL of CFS- LpMYS6 and 100 μL of Fp MYS9 (10^6^ spores/mL). Fp MYS9 (10^6^ spores/mL) alone was used as control. Along the margin of wells sterile cellophane tape was placed. The plates were then incubated for 3 days at 30°C. After incubation, cellophane tapes with attached hyphae were removed carefully and washed thrice with 0.1 M PBS. The samples were fixed with 2.5% (v/v) glutaraldehyde overnight at room temperature, rinsed thrice with 0.1M sodium phosphate buffer and dehydrated in a graded series of ethanol (30% for 10 min, 50% for 10 min, 70% for 10 min, 90% for 10min and 100% for 1 h). The samples were air dried, mounted on aluminium stub using double sided carbon tape, sputter-coated with gold and visualized using a S-3400N scanning electron microscope (Hitachi, Japan).

#### 2.3.4. Conidia germination inhibition assay

The assay was performed in a 24 well microtitre plate to evaluate the inhibitory effects of Lp MYS6 and CFS-Lp MYS6 on the conidial germination of Fp MYS9. One hundred μL each of LpMYS6 (10^6^ CFU/mL) and Fp MYS9 (10^6^ spores/mL) were mixed and made up to 1.0 mL using 0.1M PBS. Another reaction consisted of 200 μL of CFS-Lp MYS6 and 100 μL of Fp MYS9 (10^6^ spores/mL) mixed and made up to 1.0 mL using PBS. A 100 μL of Fp MYS9 (10^6^ spores/mL) made up to 1.0 mL using PBS was maintained as control. The microtitre plate was incubated for 24 h at 28°C ± 2°C. The conidial germination was observed microscopically at time intervals of 2, 4, 8, 16 and 24 h. The reaction sample was stained with Acridine Orange and observed under Fluorescent Microscope (Carl Zeiss, Germany). Germinated conidia were counted using Hemocytometer. Per cent conidia germination was calculated using the formula: [No. of conidia germinated/ Total conidia counted] ×100 [32].

#### 2.3.5. Maize-kernel deterioration assay

Maize-kernel deterioration assay was performed according to Yang and Chang [33] with slight modifications. Maize kernels were soaked in sterile distilled water for 3 h and autoclaved at 121°C for 20 min. Then these were soaked in filter sterilized CFS-Lp MYS6 for 8 h at room temperature. The maize kernels were then transferred to sterile Petri plates. Aliquot containing 20 μL of Fp MYS9 (10^6^ spores/mL) suspension was inoculated on each maize kernel and incubated at 28°C ± 2°C for 7 days. Maize kernels without CFS treatment were used as control. The fungal growth on the kernels was examined microscopically every day up to 7 days.

#### 2.3.6. Fumonisin biosynthesis inhibition in poultry feed model

The inhibitory effect of Lp MYS6 and its metabolites on fumonisin biosynthesis was analyzed using the protocol of Dalie et al. [34] with minor modifications. Culture tubes containing 5 g poultry feed mixture (a_w_ = 1, autoclaved at 121°C for 20 min) were inoculated with 100 μL of Fp MYS9 (10^6^ spores/mL) and 100 μL of LpMYS6 (10^6^ CFU/mL). Control consisted of 5 g of feed mixture inoculated with 100 μL of Fp MYS9 (10^6^ spores/mL). The culture tubes were incubated in dark for 30 days at 28°C ± 2°C and fumonisin content was measured at intervals of 4, 7, 11, 15, 21 and 30 days. To determine the effect of CFS—LpMYS6 on fumonisin biosynthesis, the same experiment was followed but 1.0 mL of CFS-Lp MYS6 was used instead of bacterial cells for the treatments. Culture tubes with only Fp MYS9 were used as control.

Fumonisins were extracted from the treated feed mixtures using acetonitrile/water (1:1) as the extraction solvent. The extract was filtered using 0.45 μm pore sized nylon membrane filters (Axiva) and subjected to liquid chromatography/mass spectrometry (LC/MS) (Waters Acquity/ Synapt G2, USA). Chromatographic separation was achieved on a C18 column maintained at 50°C. Mobile phase A was 0.3% formic acid in water (v/v) and mobile phase B was acetonitrile. The injection volume was 20 μL and the elution time was 8 min. The mass spectrometer was operated in the positive electron spray ionization mode (ESI+). The capillary voltage was set at 1.8 kV and the cone voltage was 40V. The source and desolvation temperature was 100°C and 200°C respectively and desolvation gas flow rate, 500 L/h. Helium was used as collision gas. MassLynx SCN781 software was used to validate the LC/MS results. Standard fumonisin B1 toxin (Cayman Chemical, USA) was used as the reference.

### 2.4. Fumonisin detoxification study

LpMYS6 was analyzed for its ability to bind and/or biotransform fumonisin with some modifications in the method followed by Niderkorn et al. [8]. The concentration of bacterial pellet was adjusted to 10^6^ CFU/mL using 0.1 M PBS (pH 7.4). Bacterial suspension (100 μL) was mixed with 20 μL of FB1 toxin (10 μg/mL) in a microfuge tube. The volume was made up to 1.0 mL using PBS. Positive control containing only the toxin in PBS and the negative control having only bacteria suspended in PBS were maintained. All the tubes were incubated at 37°C for 2 and 4 h. The assay was performed in duplicate. After incubation, the tubes were centrifuged (5000 rpm, 8 min, 4°C) and the supernatants were quantitatively analyzed for FB1 toxin using LC/MS. The percent toxin eliminated was determined by applying the following formula: % removal = [1- (peak area of toxin in supernatant/peak area of toxin in positive control)] × 100.

### 2.5. Extraction, purification and analysis of antifungal metabolites of *L*. *plantarum* MYS6

Antifungal metabolites were extracted from CFS-Lp MYS6 using ethyl acetate (1:3, v/v). The extracted hydrofacies were concentrated by rotary evaporator under vacuum at 55°C. The extract showed antifungal activity and hence was used for further purification. The concentrated extract obtained was separated using thin layer chromatography (TLC) performed on silica gel sheets. Two solvent systems used for the separation of metabolites were butanol, acetic acid, water (4:1:5) and chloroform, methanol (9:1) followed by detection under ultraviolet wavelength of 254 nm. The extract was evaporated to dryness, dissolved in ethyl acetate and checked for antifungal activity. Active fractions were scrapped, pooled and treated with acetone thrice. The antifungal activity of the active fraction was reconfirmed by bioautography. The composition of the active fraction was determined by GC-MS (Thermo Scientific, USA) as per the method of Sangmanee and Hongpattarakere [35]. The analysis of the active fraction was performed by a Shimadzu QP-2010 Gas Chromatograph coupled to the Shimadzu GCMSQP– 2010 Mass Spectrometer with a SGE BPX-5 column (30 m length, 0.25 μm film thickness). Helium was used as a carrier gas at a constant flow rate of 0.8 mL/min. The active fraction was dissolved in methanol and 1.0 μL of the sample was injected using AOC5000 auto injector with a split ratio 100:1. The initial temperature was set to 50°C, and then increased at a rate of 3°C /min to 280°C and held isothermally for 5 min. For MS detection, ion source temperature was set to 200°C, an electron ionization mode with ionizing energy of 70 eV and scan mass range of 100–1200 amu was employed. The compounds were identified by comparing their relative retention times and fragmentation patterns of mass spectra with those reported in the literature as well as at the National Institute of Standards and Technology (NIST) data library.

### 2.6. Statistical analysis

The data obtained in this study are the mean of triplicate determinations expressed as mean ± standard deviation and analyzed by one-analysis of variance (ANOVA) followed by Bonferroni’s multiple comparison test. The graphs were drawn using Graph pad Prism version 5.03 software (GraphPad Software Inc.).

## 3. Results and Discussion

### 3.1. Fungal identification and its ability to produce fumonisin

Contamination of animal and poultry feeds by mycotoxigenic fungi and accumulation of toxins during pre-harvest and post-harvest stages have attracted the attention of scientific and economic world due to their impact on human and animal health, animal productivity and performance as well as on trade business. Fungal infestation and mycotoxin contamination represent a serious problem not only due to the unpalatability of contaminated feed but also because of the reduction in feed quality, organoleptic attributes and nutritional properties. In this study, a total of 108 animal/poultry feeds were collected from different districts of Karnataka, India. The preliminary identification was done based on the fungal manual and keys [36]. The frequency, relative density and percent infection of feed mixtures associated with *F*. *proliferatum* were determined (data not shown). PCR screening using species specific primers has identified the fungus as *F*. *proliferatum*. The fungus also harbored the *fum* gene responsible for fumonisin synthesis. The sequence information has been submitted to Genbank under the accession number KJ159072. The production of fumonisin toxin by Fp MYS9 was confirmed by LC/MS and the concentration of the toxin was found to be 617.5 mg/ Kg of feed (S1 Fig).

### 3.2. Isolation, identification and characterization of LAB

A growing need for the potent probiotic strains in food/feed industries, health oriented products and also in combating human and animal pathogens has stimulated immense research interest in LAB.

An attempt has been made to isolate and characterize potent strains of LAB from fermented product for its possible application as an antifungal agent. A total of 11 LAB were isolated from the home-made pomegranate wine. Among the 11 LAB, the isolate which exhibited potent antifungal activity was selected for further studies. Presumptive tests confirmed that the isolate was rod shaped, Gram-positive and catalase negative. The isolate showed negative hydrolysis for arginine and was a homo-fermentative lactic acid bacterium producing only acid on glucose utilization without any gas production. The isolate fermented glucose, maltose, arabinose, lactose, xylose resulting in acid production but could not utilize mannitol, sorbitol and raffinose. The ideal growth of the isolate was at 37°C and 3% NaCl (S1 Table). Our lab earlier reported different isolates of LAB from sorghum-based traditional fermented food with probiotic attributes [21].

Our LAB isolates exhibited varying degrees of fungal inhibition against Fp MYS9 in agar overlay method. While in well diffusion assay, only two isolates showed inhibition zones around the wells provided with 250 μL or 500 μL of the CFS of the cultures (S2 Fig). Previous studies have endorsed the use of these methods for the preliminary identification of antifungal activities of LAB [22, 37]. The isolate which exhibited substantial antifungal activity in both overlay and well diffusion assays was used for further probiotic characterization.

**Fig 1:**
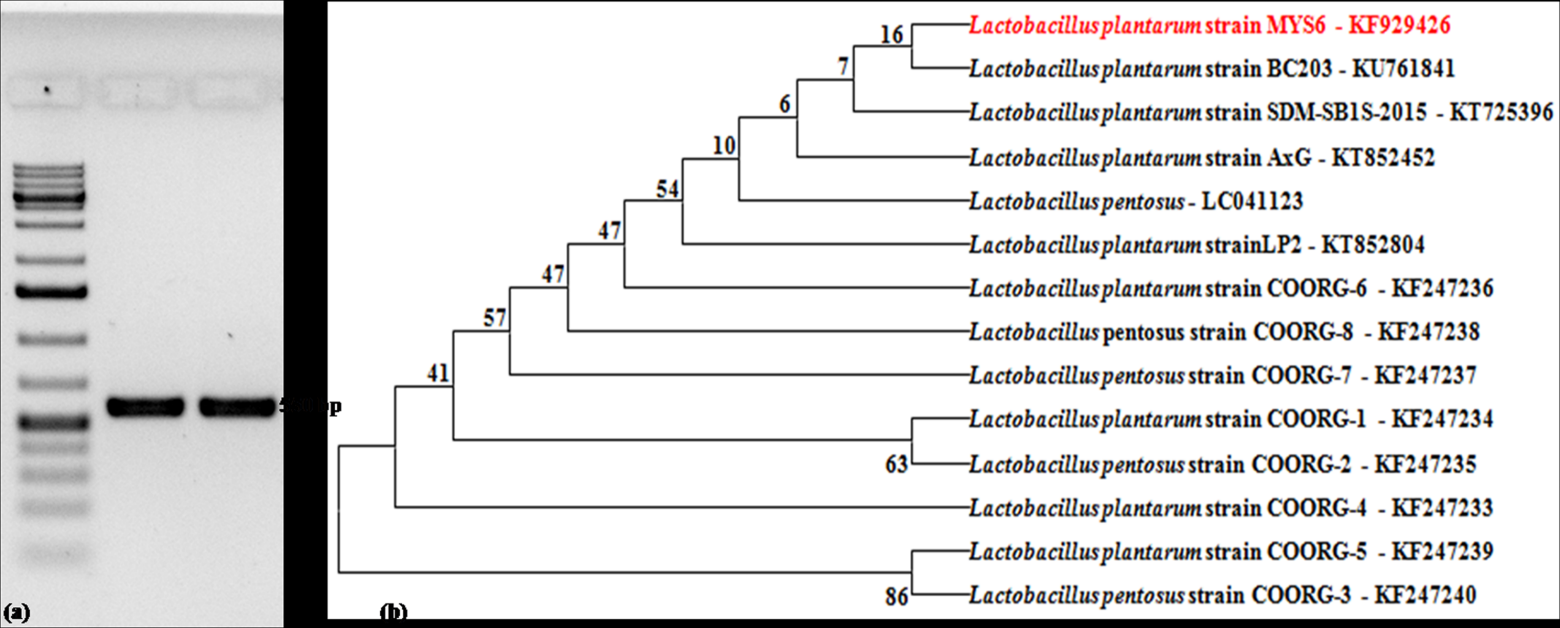
Molecular identification and phylogenetic analysis of the LAB. (a) Agarose gel showing amplicon (~550 bp) of 16S rRNA gene of the isolate; lane M–Marker, 100 bp ladder, lane 1 –*L*. *plantarum*MTCC 9483, lane 2—*L*. *plantarum* MYS6, (b) Phylogenetic tree based on the 16S rRNA gene sequence showing relationship between different *Lactobacillus* species. The dendrogram was constructed based on the BLAST algorithm and neighbour joining method.

16S rDNA sequence analysis of the isolate was represented in the dendrogram based on BLAST algorithm and compared with our previously published other isolates of *Lactobacillus* [21] and also related to *Lactobacillus* sequences deposited in Genbank (Fig 1). The 16S rDNA sequence of the isolate (*L*. *plantarum* MYS6) has been assigned the accession number KF929426 by Genbank.

Characterization of *Lactobacillus* for probiotic attributes is a necessary selection criterion to consider the isolate as ‘GRAS’ (generally regarded as safe). Tolerance of a LAB to acidic pH of gastrointestinal tract (GIT) is an essential attribute of a probiotic strain. The strain should survive the transit through proventriculus, gizzard and the upper region of the intestine before reaching the distal end of intestinal tract, where LAB exerts its probiotic effect. The pH of the GIT of poultry ranges from 2.5 to 4.7 and ingestion can account up to 1–3 h based on the size of feed. In the present study, LpMYS6 thrived well at pH 3.0 and 6.0 for 3 h of incubation with less reduction in the cell count (Table 1). Though the isolate survived initially at pH 2.0, substantial decrease in cell viability was observed with increase in incubation time (Table 1). Salah et al. [25] reported that only 4 strains among 100 LAB isolated from the gastrointestinal tract of poultry survived at pH 3.0. In another study by Musikasang et al. [38], resistance and survival at pH 3.0 was shown by only 6 isolates out of 20 LAB isolated from chicken and hence the feature is considered as strain dependant.

**Table 1:** Probiotic attributes of *L*. *plantarum* MYS6.

Tests	*L*. *plantarum* MYS6		
**Tolerance to acidic pH**	^a^**log CFU mL**^**-1**^		
	**0h**	**1h**	**3h**
**pH 2**	6.04±2.42	4.24±1.21	2.96±1.42
**pH 3**	6.16±1.42	5.26±2.42	4.42±1.42
**pH 6**	6.19±2.42	6.02±1.42	5.98±2.41
**Antibiotic susceptibility**	**Susceptible**	**Resistant**	
	Vancomycin	Kanamycin	
	Penicillin G	Gentamicin	
	Bacitracin	Norfloxacin	
	Amoxycillin		
	Erythromycin		
	Chloramphenicol		
**Hemolytic test**	Negative		
**Cell Surface Hydrophobicity**			
	**n-Hexadecane**^a*^	**Xylene**^b*^	
*Lactobacillus plantarum* MYS6	64.18±2.4	56.89±1.6	
*Lactobacillus plantarum* MTCC 9483	56.80±2.2	55.60±1.6	

n = 3

^a^ Mean±SD of log value of cell viability

^a*^ Mean±SD of % hydrophobicity

^b*^ Mean±SD of % hydrophobicity

**Fig 2:**
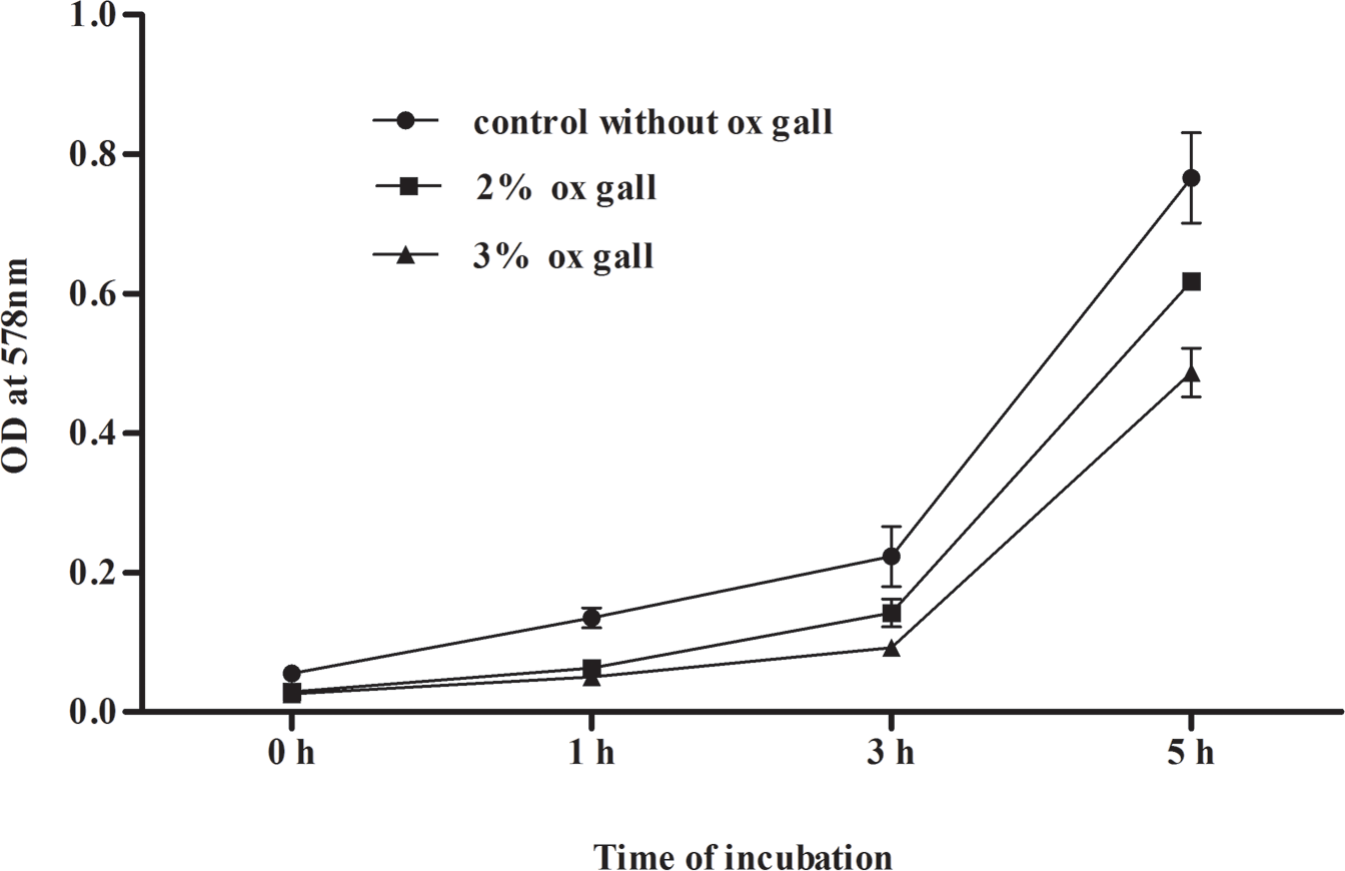
Growth of *L*. *plantarum* MYS6 in the presence of 2 and 3% ox gall at different time intervals.

The bile salt in the intestine is a key factor affecting the viability and growth of LAB. Accordingly, growth and tolerance study of LpMYS6 at 2% and 3% ox gall (bile salt) for 5 h was conducted and results are illustrated in Fig 2. The isolate exhibited strong tolerance to bile as evidenced by similar pattern of growth as that of positive control without bile. Our results are in consistent with the reports of Park et al. [39] according to which the growth kinetics of *L*. *plantarum* KCTC3179 was slightly affected even in 5% concentration of ox gall. Rao et al. [21] showed viability of *L*. *plantarum* and *L*. *pentosus* strains by hydrolyzing 0.3% ox gall. Salah et al. [25] reported that *L*. *plantarum* strains TN8, TN1 and TN13 grew well in 3 and 5% bovine bile. It is important to know as a probiotic the susceptibility of LAB to antibiotics, as transmissible mechanisms of resistance can have serious consequences. The susceptibility and resistance of the isolate Lp MYS6 to different antibiotics is shown in Table 1. Hemolytic activity refers to the breakdown of the red blood cells and it is an indication of bacterial virulence. Thus absence of hemolysis is one of the attributes of probiotic strain. In the present study, LpMYS6 showed no-haemolysis (S3 Fig) on a blood agar plate. The cell surface hydrophobicity of the isolate was also measured by its adhesion capacity to n-hexadecane and xylene which are non-polar. LpMYS6 showed high hydrophobicity of 64.18% towards n-hexadecane and 56.89% towards xylene as compared to the standard strain, *L*. *plantarum* MTCC 9483 (Table 1). This indicates the strong electron donating property of the isolate and is on par with the control.

**Fig 3:**
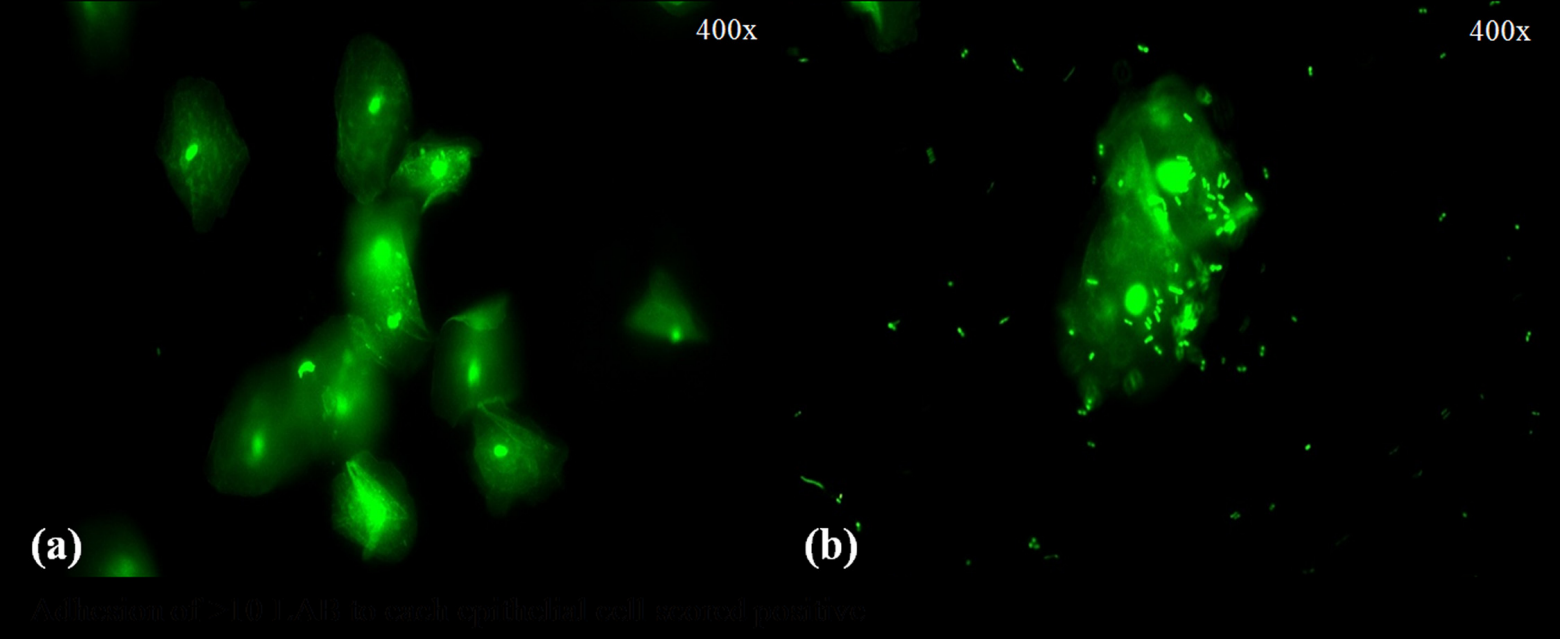
Adhesion of *L*. *plantarum* MYS6 to chicken crop epithelial cells (a) control-epithelial cells (b) adherence of *L*. *plantarum* MYS6 to epithelial cells.

LpMYS6 exhibited strong adhesive property to the chicken crop epithelial cells as evidenced by the attachment of 10–15 bacteria/epithelial cells (Fig 3). Variations in the adhesion properties of LAB to epithelial lining are mainly due to differences in bacterial cell wall composition and are strain specific [40]. In a study by Ehrmann et al. [26], *L*. *reuteri* TMW, *L*. *salivarius* TMW, and *L*. *animalis* TMW scored positive as they adhered approximately ten bacteria to crop epithelial cells. Also in a study by Salah et al. [25] *L*. *plantarum* TN8 isolated from poultry gizzard showed effective adhesion to the chicken enterocytes of duodenum (82%), jejunum (84%) and ileum (70%). Therefore the isolate Lp MYS6 showed high cell surface hydrophobicity and strong adhesion to crop epithelial cells thereby proving probiotic nature.

### 3.3. Antifungal activity of *L*. *plantarum* MYS6

A modified MRS medium was designed to support the growth of both LAB and fungi. In this medium, biomass of the fungus (Fp MYS9) and log CFU mL^-1^ of Lp MYS6 was observed in 3, 7, 10 and 14 days (Table 2). There was a slow kinetics in the fungal growth and biomass formation in presence of LpMYS6. In the 14-day old control culture, the biomass was 1.65g while in the treated cultures it was 1.5g. Lp MYS6 survived throughout the incubation period but a decrease in the cell viability was observed with the increasing incubation time (Table 2). A similar study performed by Dalie et al. [34] reported slow growth and biomass accumulation of9.18 mg/mL by *Fusarium verticillioides* in the presence of *Pediococcus pentosaceus* strain L006 in GYEAP medium as compared to the control which produced dry biomass of 12.05 mg/mL. But interestingly there was also increase in fumonisin production which could be due to stressful environment induced by *P*. *pentosaceus*. But our study concentrated only on the inhibition of fungal growth and biomass by the LAB and did not assay the fumonisin production. Nevertheless, our’s is an attempt of its first kind in exploring the antifungal potential of lactobacilli against *F*. *proliferatum* which is the second most significant producer of fumonisins.

**Table 2:** Effect of *L*. *plantarum* MYS6 on biomass production by *F*. *proliferatum* MYS9 co-inoculated on modified MRS medium.

	Biomass (g)		log CFU mL^-1^	
Days	Fp MYS9	Fp MYS9 + Lp MYS6	Lp MYS6	Fp MYS9 + Lp MYS6
**3**	0.493 ± 0.13	0.309 ± 0.07	8.002 ± 0.01	7.993 ± 0.00
**7**	0.928 ± 0.20	0.752 ± 0.08	7.916 ± 0.00	7.897 ± 0.00
**10**	1.261 ± 0.16	1.093 ± 0.15	7.815 ± 0.01	7.789 ± 0.00
**14**	1.658 ± 0.06	1.503 ± 0.25	4.533 ± 0.03	4.487 ± 0.06

Mean±SD; Fp MYS9—*F*. *proliferatum* MYS9; Lp MYS6 –*L*. *plantarum* MYS6

**Fig 4:**
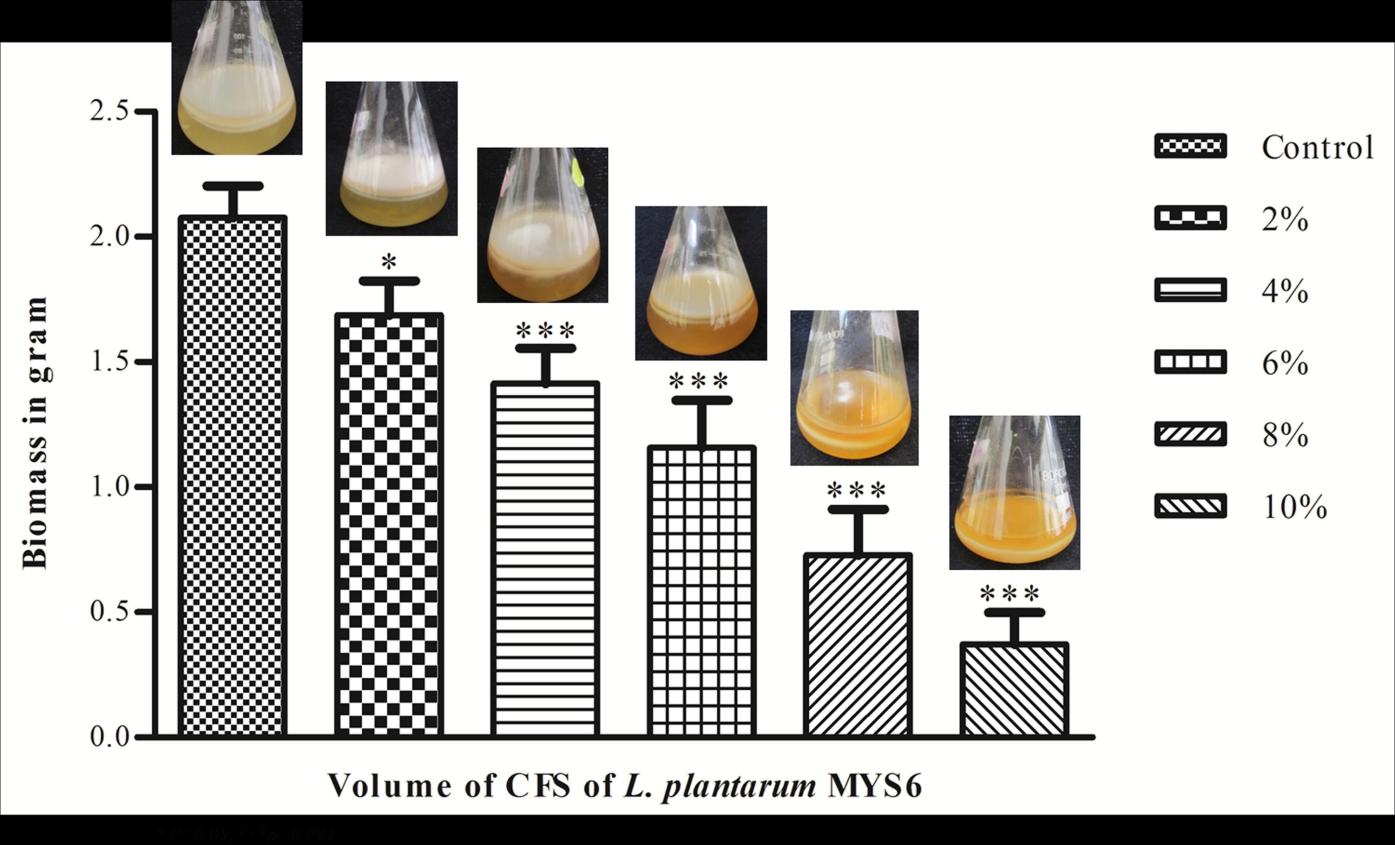
Effect of CFS of *L*. *plantarum* MYS6 on mycelial biomass of *F*. *proliferatum* MYS9.

We also observed a great reduction in fungal biomass production when treated with the cell free supernatant of Lp MYS6 (Fig 4). This could be due to the metabolites secreted by LpMYS6 into the medium. Incubation of control for 20 days yielded a biomass of 2.07 g while a progressive reduction in mycelial growth of 1.686, 1.413, 1.158, 0.728 and 0.372 g was observed in cultures treated with 2, 4, 6, 8 and 10% CFS, respectively. Earlier study by Arasu et al. [31] demonstrated the antifungal activity of the cell-free supernatant of *L*. *plantarum* KCC-10 against different fungi (*Aspergillus niger*, *A*. *fumigatus*, *Candida albicans*, *F*. *verticillioides*, *F*. *solani*, *Penicillium chrysogenum*, *Botrytis elliptica* etc.,) and maximum growth inhibition was recorded by *B*. *elliptica* (79.58%) followed by *A*. *fumigatus* (67.3%). Another study conducted by Strom et al. [41] described the inhibitory effect of metabolites produced by *L*. *plantarum* MiLAB 393 which decreased the biomass formation of *Aspergillus nidulans* to 36% of the control.

**Fig 5:**
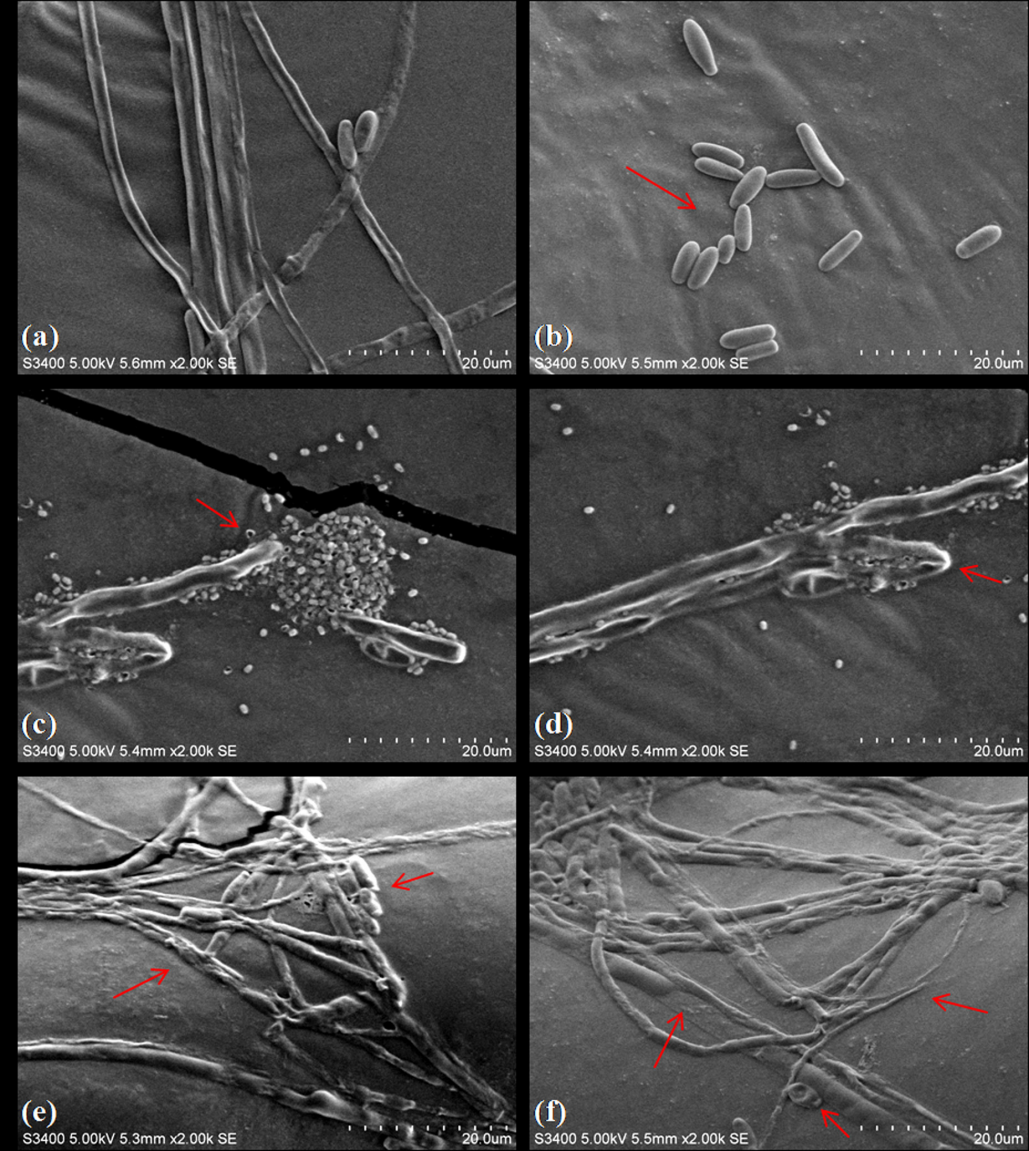
Scanning electron micrographs showing the effects of *L*. *plantarum* MYS6 and its CFS on *F*. *proliferatum* MYS9. (a, b) Control, *F*. *proliferatum* MYS9 appears to be tubular, even width, smooth surfaced hyphae and conidia showing a typical club shape-flattened base, (c, d) *F*. *proliferatum* MYS9 treated with *L*. *plantarum* MYS6 showing disrupted, wrinkled hyphae with swollen tips, (e, f) *F*. *proliferatum* MYS9 treated with CFS of *L*. *plantarum* MYS6 showing deformed, flattened and shrunken hyphae bearing disrupted conidia. Images obtained by scanning electron microscopy at 2000x magnification.

SEM analysis provided the effect of Lp MYS6 and its antifungal metabolites on the morphology of hyphae and conidia. The control had regular hyphal growth displaying tubular, even width, smooth surfaced and elongated structures. Conidia showed a typical club shape and flattened base (Fig 5A and 5B). In contrast, Lp MYS6 treatment resulted in aberrant and distorted hyphal structures. The hyphae were wrinkled with conglobated tips. Also, the bacteria were in collective mass around the hyphae disrupting the fungal structure (Fig 5C and 5D). CFS-Lp MYS6 also caused substantial deformation, uneven width and damage to mycelia. Flattened and twining hyphae, shrunken and wrinkled hyphal surface were conspicuous. The conidial size was reduced and surface was disrupted (Fig 5E and 5F). Alterations in the hyphal and conidial structures correlated with conidial germination inhibition assay. Sangamanee and Hongpattarakere [35] showed severe damage and distortion of hyphal structures of *Aspergillus flavus* and *A*. *parasiticus* on exposure to 5.87 mg/mL of supernatant of *Lactobacillus plantarum* K35. Gong et al. [42] showed the antagonistic property of Iturin A and Plipastatin A from *Bacillus amyloliquefaciens* against *Fusarium graminearum*. These compounds damaged fungal hyphae and inhibited conidial germination at a concentration of 50 μg/mL (Iturin A) and 100 μg/mL (Plipastatin A). Our results also indicate that Lp MYS6 and its antifungal metabolites damage structural integrity of fumonisin producing *F*. *proliferatum* MYS9 and interfere with fungal cell development.

**Fig 6:**
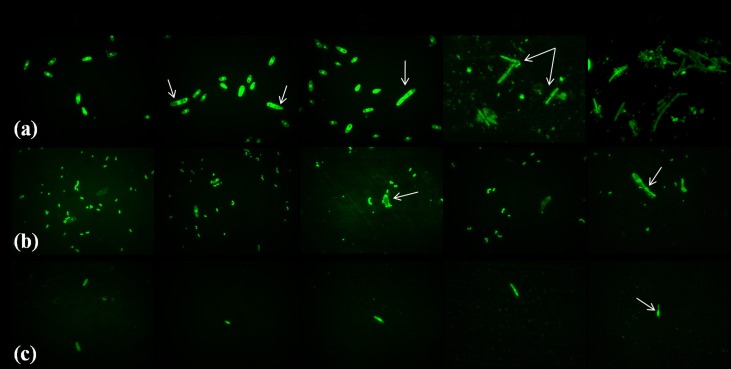
Conidia germination inhibition of *F*. *proliferatum* MYS9 by*L*. *plantarum* MYS6 and its CFS. (a) Control, *F*. *proliferatum* MYS9 showing germ tube initiation and formation of regular, hyphae,(b) *F*. *proliferatum* MYS9 treated with *L*. *plantarum* MYS6 showing distorted germ tube formation, (c) *F*. *proliferatum* MYS9 treated with CFS of *L*. *plantarum* MYS6 showing retarded germination and hypahl growth. Images obtained by Fluorescent microscopy at 1000x magnification.

The conidia germination inhibition assay exhibited a prominent inhibition of fungal growth. The *Lactobacillus* isolate and its metabolites affected conidial germination and mycelial development. Microscopic observation did not reveal any germination in the initial 2 h incubation in the control and treatment samples. The per cent conidia germination is given in Table 3. Fp MYS9 (control) developed germ tube from the apical cells after 4 h of incubation recording 32.04% conidia germination (Fig 6A). No apical cell elongation was observed in the treated samples after second interval of incubation period. After 8 h of incubation, appreciable outgrowth of germ tubes was visible in the control sample. But conidia treated with Lp MYS6 showed retardation in germination and the bacteria were adhering on to the conidia (Fig 6B). In Fp MYS9 treated with CFS- Lp MYS6, germ tube emerged from non-apical cells of the conidia and showed uneven outgrowth (Fig 6C). After 24 h of incubation, there was complete germination in all conidia in the control, exhibiting linear, well grown and intact hyphal structures. Distorted germ tube formation was more frequent in Fp MYS9 exposed to Lp MYS6 and has resulted in irregular and unorganized hyphal growth (Fig 6B). Antifungal metabolites present in CFS-Lp MYS6 created a stressful environment hampering conidial germination and hyphal outgrowth significantly (p<0.05) after 24 h of incubation with only 19.58% of germination. Mauch et al. [43] also investigated the antifungal activity of *Lactobacillus brevis* PS1 against macroconidia germination and mycelial growth of *Fusarium culmorum*. Treatment with 5% cell free supernatant of *L*. *brevis* PS1 (cfsP) resulted in macroconidia possessing more than two germ tubes or emergence of germ tube from internal components with a disrupted mycelial outgrowth. While 10% cfsP completely restricted the germination of macroconidia. Our observations on the hyphal morphology were similar to Koch and Loffler [44] who reported disrupted and disorganized hyphae when treated with antifungal filtrate of *Streptomyces antimycoticus*.

**Table 3:** Conidia germination inhibition by *L*. *plantarum* MYS6.

	% conidia germination		
Time h	Fp MYS9	Fp MYS9 + Lp MYS6	Fp MYS9 + CFS-Lp MYS6
**2**	0.0	0.0	0.0
**4**	32.05 ± 1.11	0.0	0.0
**8**	54.71 ± 4.16	15.88 ± 2.26	7.85 ± 2.40
**16**	74.17 ± 3.25	33.94 ± 2.33	15.08 ± 2.26
**24**	100.0 ± 0.00	49.01 ± 1.69	20.32 ± 1.75

Mean±SD; Fp MYS9—*F*. *proliferatum* MYS9; Lp MYS6 –*L*. *plantarum* MYS6; CFS-Lp MYS6 –cell free supernatant of *L*. *plantarum* MYS6.

**Fig 7:**
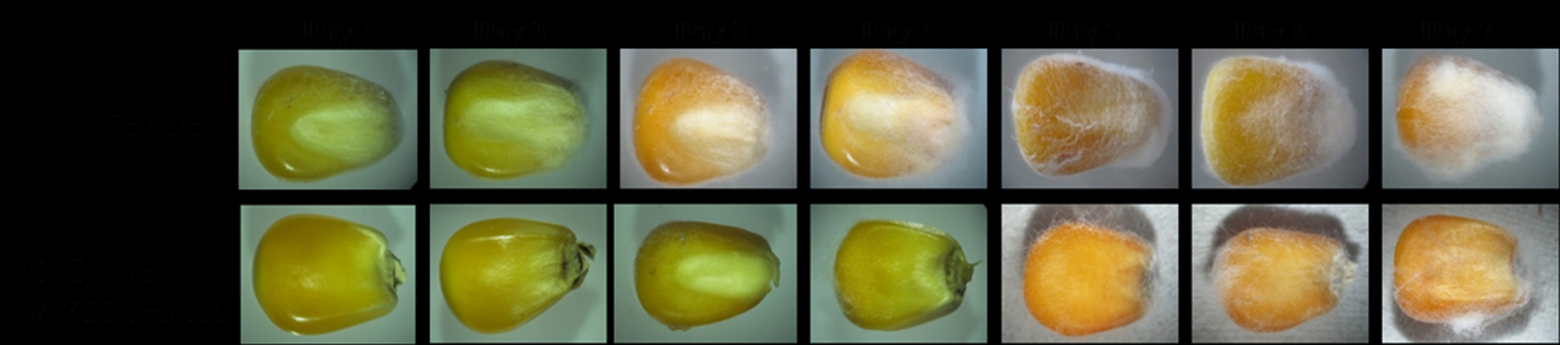
Effect of CFS of *L*. *plantarum* MYS6 on maize kernels inoculated with *F*. *proliferatum* MYS9.

Maize is a major ingredient used in the formulation of poultry feed mixtures. Protection of maize grains from fungal damage is important for the enhanced shelf-life of feed mixtures. In this regard, maize kernel deterioration assay was performed to study the potential application of antifungal metabolites of LpMYS6 to overcome fungal spoilage of stored food/feed grains. In control, white mycelia were observed from day 2 of incubation and almost covered the kernel in 7 days. But maize kernels treated with CFS-Lp MYS6 had no growth of Fp MYS9 up to 4 days and partial inhibition of fungal growth was observed afterwards (Fig 7). The ability of CFS-Lp MYS6 to prevent the growth of *F*. *proliferatum* on maize-kernels suggests its antifungal property. Yang and Chang [33] used soybeans for analyzing the antifungal activity of *L*. *plantarum* isolated from a fermented kimchi against *Aspergillus flavus*. They attributed this property to a new antifungal compound, 3,6-bis(2methylpropyl)-2,5-piperazinedion which could be a promising alternative to chemical preservatives. No previous studies have reported the antifungal as well as biopreservative activity of *L*. *plantarum* against fumonisin producing *F*. *proliferatum*.

By using poultry feed mixture of maize, we investigated the influence of co-inoculation of LpMYS6 and Fp MYS9 and treatment with cell-free supernatant of Lp MYS6 on fumonisin biosynthesis. When co-inoculated and tested in feed, no fumonisin was detected in the control and in treated samples up to 15 days after incubation. However, in the control, fumonisin content of 0.8425 mg/Kg feed and 0.870 mg/Kg feed were found on 21 and 30 days of incubation respectively. While in the treated samples, fumonisin content of 0.475mg/Kg feed and 0.505 mg/Kg feed were detected on 21 and 30 days after incubation. The reduction was 53.8% in fumonisin content in the treated samples when compared to the control. Treatment of the feed with CFS-Lp MYS6 showed a complete absence of the toxin at 21 days incubation. A reduction of 0.3125 mg/Kg feed (73%) in fumonisin content at 30 days-culture was observed in treated sample (Table 4, S4 Fig). Our data suggest that the possible role of metabolites produced by LpMYS6 in efficiently suppressing fumonisin production by Fp MYS9. Study conducted by Dalie et al. [34] using maize has shown no significant effect on fungal growth and fumonisin production by *F*. *verticillioides* when co-inoculated with *Pediococcus pentosaceus* L006. But maize-kernels treated with concentrated supernatant of bacterial culture showed a significant reduction in growth of *F*. *verticillioides* (50%) as well as in fumonisin production (75%) by the fungus. Present study has shown that fumonisin production was significantly hampered by co-inoculation with LpMYS6 and treatment with its CFS.

**Table 4:** Effect of *L*. *plantarum* MYS6 and CFS of *L*. *plantarum* MYS-6 on fumonisin biosynthesis by *F*. *proliferatum* MYS9 cultured in poultry feed up to 30 days.

	Fumonisin biosynthesis (mg/Kg feed)		
Days	Fp MYS9	Fp MYS9 + LpMYS6	Fp MYS9 + CFS-LpMYS6
**4**	ND	ND	ND
**7**	ND	ND	ND
**11**	ND	ND	ND
**15**	ND	ND	ND
**21**	0.842 ± 0.12	0.475± 0.05	ND
**30**	0.870 ± 0.10	0.505± 0.01	0.3125± 0.04

ND-not detected; Mean ± SD; Fp MYS9 –*F*. *proliferatum* MYS9; Lp MYS6 –*L*. *plantarum* MYS6; CFS-Lp MYS6 –cell free supernatant of *L*. *plantarum* MYS6.

Based on these studies, *L*. *plantarum* MYS6 and its cell free supernatant seem to be a promising biocontrol agent for inhibiting *F*. *proliferatum* and also to reduce fumonisin contamination in feed. It is also important to note that the presence of Lp MYS6 in the medium or in the feed did not induce a stressful environment that might favor toxin production.

### 3.4. Fumonisin detoxification study

The ability of LAB to bind or biotransform fumonisin is a promising alternative to the physical and chemical detoxification methods widely employed in food/feed industries. It will also reduce the bioavailability and toxic effects of fumonisin to human and poultry. In the present study we made an attempt to understand the mechanism of detoxification of fumonisin by Lp MYS6 through LC/MS analysis. This revealed that fumonisin was not transformed as peaks corresponding to the aminopentol derivatives of the toxin were not detected in 2 and 4 h of incubation. But fumonisin content was found to decrease with incubation time. Moreover, there were no peaks which represented the degradation products of fumonisin. The per cent removal of fumonisin was 32.9% in the initial 2 h of incubation while it was 61.7% in 4 h showing a substantial reduction in fumonisin content (Table 5). Similar experiments conducted by Niderkorn et al. [8, 9] on detoxification of fumonisins (FB1 and FB2) by *Lactobacillus*, *Leuconostoc*, *Pediococcus*, *Propionibacterium* etc. did not show any derivatives of fumonisins, though the removal of fumonisins was conspicuous after 24 h of incubation. This was attributed to the binding ability of LAB to fumonisins as no degradation residues could be observed in the HPLC chromatogram. They observed that *Streptococcus* and *Enterococcus* are the most efficient LAB, as the binding was 24% and 62% fumonisins respectively. Further they studied on the binding ability of LAB and propionic acid bacteria to fumonisins and reported 82% and 100% removal of FB1 and FB2 respectively by LAB, but propionic acid bacteria were less efficient in removing the toxin. There are also reports on the binding ability of different strains of *Lactobacillus rhamnosus* to aflatoxin B and trichothecenes [1, 45], and *Propionibacterium* to trichothecenes [11]. Niderkorn et al. [46] suggested that the peptidoglycan of LAB and tricarballylic acid arm of FB1 play a significant role in binding. Based on these studies, it is tempting to speculate that the isolate Lp MYS6 detoxifies fumonisin by some binding mechanisms. However, further detailed investigation on the interaction mechanism and sustainability has to be carried out for practically exploiting the LAB in feed industries. To understand the potency of LAB in restraining intestinal toxin absorption using *in vivo* models of poultry/livestock is our future perspective.

**Table 5:** Fumonisin removal by *L*. *plantarum* MYS6.

Time h	Toxin control^†^	Treatment^†a^	Fraction removed (%)
**2**	1427.82	958.205 ± 39.87	32.9
**4**	1533.64	588.390 ± 77.81	61.7

† peak area

†^a^ mean±SD peak area

### 3.5. Analysis of antifungal metabolites

**Fig 8:**
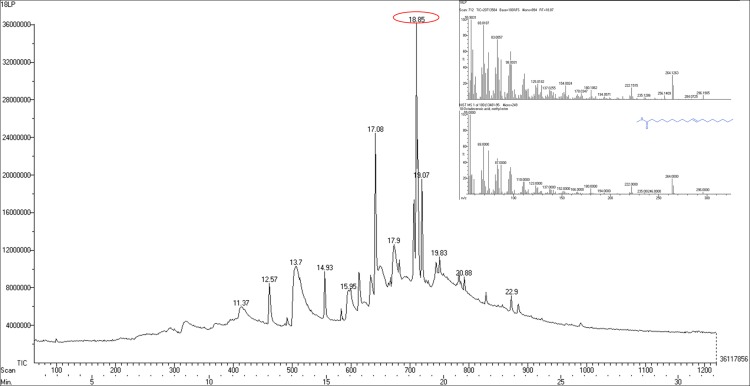
GC/MS analysis of CFS of *L*. *plantarum* MYS-6.

Liquid-liquid extraction of CFS-Lp MYS6 with ethyl acetate generated organic and aqueous phases, in which, the antifungal metabolites were largely concentrated in the latter facet. The butanol: acetic acid: water fraction revealed no antifungal activity whereas the chloroform: methanol fraction possessed antifungal property against Fp MYS9 (S5 Fig). The GC/MS analysis of CFS-Lp MYS6 revealed 12 compounds (Fig 8). LpMYS6 produced multiple antifungal compounds which are free long chain fatty acids and fatty acid esters, having chain length ranging from 12 to 23 carbons. The compounds shared the common feature of being small molecules with molecular mass ranging from 162.14 g/mol to 354.61 g/mol (Table 6). The sequential order of major compounds were 10-Octadecenoic acid, methyl ester at 18.85 min, hexadecanoic acid, methyl ester (palmitic acid, methyl ester) at 17.08 min, heptadecanoic acid, 16-methyl ester at 19.07 min, octadecanoic acid (stearic acid) at 17.9 min and dodecanoic acid (Lauric acid) at 13.7 min retention time. Apart from the fatty acids and fatty acid esters, a cyclic compound namely 6-deoxy-d mannono-4-lactone at 11.37 min was also detected (S6 Fig).

**Table 6:** Chemical composition of CFS of *L*. *plantarum* MYS6 by GC/MS analysis.

Retention Time min	Compound	Molecular mass g/mol	PubChem CID
11.37	6-Deoxy-D-mannono-4-lactone	162.14	242516
12.57	Undecanoic acid, 10-methyl, methyl ester	214.34	554144
13.7	Dodecanoic acid	200.31	3893
14.93	Methyl tetradecanoate	242.39	31284
15.95	Tetradecanoic acid	228.38	11005
17.08	Hexadecanoic acid, methyl ester	270.45	8181
17.9	Octadecanoic acid	284.47	5281
18.85	10- Octadecenoic acid, methyl ester	296.48	5364425
19.07	Heptadecanoic acid, 16-methyl, methyl ester	298.50	8201
19.83	6-Octadecenoic acid (Z)	282.46	5282754
20.88	Nonadecanoic acid, 18-oxomethyl ester	326.51	536994
22.9	Docosanoic acid, methyl ester	354.61	13584

Generally, a broad spectrum of antifungal carboxylic acid esters is produced by lactic acid bacteria [31, 35, 37] which is species and strain dependent. The antifungal efficiency of saturated or unsaturated free fatty acids increases with increase in chain length. In the present study, LpMYS6 produced long chain fatty acids and fatty acid esters. Among them saturated compounds were C_12_ to C_15_, C_17_, C_20_ and C_23_; saturated and unsaturated were C_18_ and C_19_. 10-Octadecenoic acid, methyl ester, is the most predominant compound produced by Lp MYS6 and it is an unsaturated C_19_ fatty acid ester which has not been so far reported as an antimicrobial compound. There are reports about its derivatives and analogs from plant extracts possessing antimicrobial, antioxidant and antitumor activities [47]. Masui et al. [48] reported inhibition of spore germination of *Cerotocystis fimbriata* by 9,12,13-trihydroxy-(E)-10-octadecenoic acid isolated from the tubers of *Colocasia antiquorum* and also suggested that it is produced from linolenic acid or linoleic acid by *in vivo* peroxidative reaction. The other antifungal compounds are palmitic acid, methyl ester; heptadecanoic 16-methyl, methyl ester; stearic acid; lauric acid and tetradecanoic acid (myristic acid). Similar fatty acid composition was reported from the leaves of *Excoecaria agallocha* which also exhibited antibacterial and antifungal activity [49]. Sangamanee and Hongpattarakere [35] reported the synergistic-inhibitory activity of lactic acid, 2-butyl-4-hexyloctahydro-1H-indene, phenyllactic acid, palmitic acid, stearic acid, oleic acid, linoleic acid etc. produced by *L*. *plantarum* K35 against *Aspergillus* species. Lactic acid and phenyllactic acid were not secreted by LpMYS6. Nevertheless, the antifungal metabolites secreted by Lp MYS6 showed significant antifungal activity. LpMYS6 also produced fatty acid esters namely, 6-octadecenoic acid (Z); undecanoic acid, 10-methyl, methyl ester; nonadecanoic acid, 18-oxomethyl ester, docosanoic acid, methyl ester in relatively lower amount. 6-deoxy-D-mannono-4-lactone, a cyclic ester of hydroxyl carboxylic acid, was also found in low concentration. This cyclic ester was reported by Bhogireddy et al. [50] as a phytoconstituent of methanol (100%) fraction of *Entada pursaetha* DC seeds. The fraction showed anti-inflammatory activity but the sole action of the cyclic ester was not documented.

Our study identified a range of antifungal metabolites from *Lactobacillus plantarum* MYS6, which included low-molecular weight compounds as well as combination of saturated and unsaturated fatty acids. The significant antifungal activity of LAB against *Fusarium proliferatum* MYS9 could be attributed to these metabolites. The study also reiterates the diverse distribution of antifungal substances among different strains of *L*. *plantarum*.

## Conclusion

*L*. *plantarum* MYS6 is having potent probiotic attributes and antifungal activity against fumonisin producing *F*. *proliferatum* MYS9. It is efficient in adhering to the chicken crop epithelial cells. Because of its antifungal effects and toxin binding capacity, the isolate will have wide application as a potential probiotic and biocontrol agent in feed supplements and poultry industry. Additionally, the present study also showed effective antifungal attributes by CFS-Lp MYS6 than bacterial cells in controlling conidia germination, distorting the morphology of hyphae and conidia. Many antifungal metabolites were identified in the cell-free supernatant of *L*. *plantarum* MYS6 which could be used as a promising bio-alternative to the chemical preservatives in poultry feeds.

## Supporting Information

S1 Fig Morphological and molecular identification of *F*. *proliferatum* MYS9 (a) Colony morphology,aborse, (b) reverse, (c) micromorphological features showing polyphialides and short conidial chains, (d) Agarose gel showing species specific amplicon size of ~230 bp of *F*. *proliferatum* MYS9; M- 100bp DNA ladder, lane 1 –MTCC standard *F*. *proliferatum* strain 286, lane 2—*F*. *proliferatum* MYS9 (e) Mass spectrometric and liquid chromatogram results confirming fumonisin production by *F*. *proliferatum* MYS9 at a retention time 2.18 min having a molar mass of 722 g/mol.
(TIFF)

S2 Fig Prelimenary antifungal assays. (a) Agar overlay method; a clear inhibition zone is displayed around the two streaks of *L*. *plantarum* MYS6 thus showing growth inhibition of *F*. *proliferatum*MYS9, (b) Well diffusion method; an evident inhibition of fungal growth increasing with concentration of CFS of *L*. *plantarum* MYS6.
(TIF)

S3 Fig *L*. *plantarum* MYS6 showing anti-hemolytic activity. (TIF)

S4 Fig Fumonisin biosynthesis inhibition in poultry feed model (a) control, *F*. *proliferatum* MYS9 growth up to 30 days (b) control-mass spectrum of FB1 at 21 and 30 days respectively (c) effect of *L*. *plantarum* MYS6 on *F*. *proliferatum* MYS9 growth up to 30 days (d) *L*. *plantarum* MYS6 treated -mass spectrum of FB1 at 21 and 30 days respectively (e) effect of CFS of *L*. *plantarum* MYS6 on *F*. *proliferatum* MYS9 growth up to 30 days (f) CFS of *L*. *plantarum* MYS6 treated—mass spectrum of FB1 at 21 and 30 days respectively.
(TIF)

S5 Fig TLC and its Bioautography of CFS of *L*. *plantarum* MYS6 (a) TLC separation of CFS in Chloroform:methanol solvent system showing three bands (b) bioautography showing significant inhibition of *F*. *proliferatum*MYS9 by TLC purified chloroform:methanol fraction of CFS of *L*. *plantarum* MYS6.
(TIF)

S6 Fig GCMS analysis and identification of multiple antifungal compounds of purified CFS of *L*. *plantarum* MYS6. (DOCX)

S1 Table Morphological, physiological, biochemical characterization of *L*. *plantarum* MYS6. (TIF)

## References

[1] The International Agency for Research on Cancer (IARC). Monographs on the evaluation of carcinogenic risks to humans: Some traditional herbal medicines, some mycotoxins, naphthalene and styrene. 2002; 82. PMC4781602 12687954

[2] Fandohan P, Gnonlonfin B, Hell K, Marasas WFO, Wingfield MJ. Natural occurrence of *Fusarium* and subsequent fumonisin contamination in preharvest and stored maize in Benin, West Africa. PLoS ONE. 2005; 99: 173–183 10.1016/j.ijfoodmicro.2004.08.012 15734565

[3] Morgavi DP, Riley RT. An historical overview of field disease outbreaks known or suspected to be caused by consumptionof feed contaminated with *Fusarium* toxins. Anim Feed Sci Technol. 2007; 137: 201–212.

[4] Marasas WFO, Riley RT, Hendricks KA, Stevens VL, Sadler TW, Waes JG, et al Fumonisins disrupt sphingolipid metabolism, folate transport, and neural tube development in embryo culture and *in vivo*: A potential risk factor for human neural tube defects among populations consuming fumonisin contaminated maize. J Nutr. 2004; 134: 711–716 15051815 10.1093/jn/134.4.711

[5] Huwig A, Freimund S, Kappeli O, Dutler H. Mycotoxin detoxification of animal feed by different adsorbents. Toxicol Lett. 2001; 122: 179–188. 11439224 10.1016/s0378-4274(01)00360-5

[6] Pearson T, Wicklow D, Pasikatan M. Reduction of aflatoxin and fuminisin contamination in yellow corn by high-speed dual-wavelength sorting. Cereal Chem. 2004; 81: 490–498.

[7] Davidson PM. Chemical preservatives and natural antimicrobial compounds In: Doyle MP, Beuchat LR, Montville TJ, editors. Food Microbiology—Fundamentals and Frontiers. 2nd Ed. American Society for Microbiology, Washington, DC 2001 p.593–627.

[8] Niderkorn V, Morgavi DP, Pujos E, Tissandier A, Boudra H. Screening of fermentative bacteria for their ability to bind and biotransform deoxynivalenol, zearalenone and fumonisins in an *in vitro* simulated corn silage model. Food Addit Contam. 2007; 24: 406–415. 17454114 10.1080/02652030601101110

[9] Niderkorn V, Boudra H, Morgavi DP. Binding of *Fusarium* mycotoxins by fermentative bacteria *in vitro*. J Appl Microbiol. 2006; 101: 849–856. 16968296 10.1111/j.1365-2672.2006.02958.x

[10] Haskard C, Binnion C, Ahokas J. Factors affecting the sequestration of aflatoxin by *Lactobacillus rhamnosus* strain GG. Chem Biol Interact. 2000; 128: 39–49. 10996299 10.1016/s0009-2797(00)00186-1

[11] El-Nizami HS, Polychronaki N, Salminen S, Mykkanen H. Binding rather metabolism may explain the interaction of two food-grade *Lactobacillus* strains with zearalenone and it’s a-zearalenol. Appl Environ Microbiol. 2002a; 68: 3545–3549. 12089040 10.1128/AEM.68.7.3545-3549.2002 PMC126820

[12] El-Nezami HS, Chrevatidis A, Auriola S, Salminen S, Mykkanen H. Removal of common *Fusarium* toxins *in vitro* by strains of *Lactobacillus* and *Propionibacterium*. Food Addit Contam. 2002b; 19: 680–686. 12113664 10.1080/02652030210134236

[13] Hassan YI, Bullerman LB. Antifungal activity of *Lactobacillus paracasei* ssp. *tolerans* isolated from a sourdough bread culture. Int J Food Microbiol. 2008; 121: 112–115. 18077044 10.1016/j.ijfoodmicro.2007.11.038

[14] Magnusson J, Ström K, Roos S, Sjörgen J, Schnürer J. Broad and complex antifungal activity among environmental isolates of lactic acid bacteria. FEMS Microbiol Lett. 2003; 219: 129–135. 12594034 10.1016/S0378-1097(02)01207-7

[15] Sathe SJ, Nawani NN, Dhakephalkar PK, Kapadnis BP. Antifungal lactic acid bacteria with potential to prolong shelf-life of fresh vegetables. J Appl Microbiol. 2007; 103: 2622–2628. 17850302 10.1111/j.1365-2672.2007.03525.x

[16] Gerez CL, Torino MI, Rollan G, Valdez GF. Prevention of bread mould spoilage by using lactic acid bacteria with antifungal properties. Food Control. 2009; 20: 144–148.

[17] Rouse S, Harnett D, Vaughan A, van Sinderen D. Lactic acid bacteria with potential to eliminate fungal spoilage in foods. J Appl Microbiol. 2008; 104: 915–923. 17976175 10.1111/j.1365-2672.2007.03619.x

[18] Sreenivasa MY, Gonzalez Jaen MT, Dass RS, Charith Raj AP, Janardhana GR. A PCR based assay for the detection and differentiation of potential fumonisin-producing *Fusarium verticillioides* isolated from India maize kernels. Food Biotechnol. 2008; 22: 160–170.

[19] Jurado M, Vazquez C, Marin S, Sanchis V, Gonzalez Jaen MT. PCR-based strategy to detect contamination with mycotoxigenic *Fusarium* species in maize. Syst Appl Microbiol. 2006 29: 681–689. 16513314 10.1016/j.syapm.2006.01.014

[20] Proctor RH, Plattner RD, Brown DW, Seo JH, Lee YW. Discontinuous distribution of fumonisin biosynthetic genes in the *Gibberella fujikuroi* species complex. Mycol Res. 2004 108: 815–822. 15446715 10.1017/s0953756204000577

[21] Rao KP, Chennapa G, Suraj U, Nagaraja H, Charith Raj AP, Sreenivasa MY. Probiotic potential of *Lactobacillus* strains isolated from sorghum-based traditional fermented food. Probiotics Antimicrob Proteins. 2015; 7: 146–156. 10.1007/s12602-015-9186-6 25666113

[22] Magnusson J, Schnurer J. *Lactobacillus coryniformis* subsp. *coryniformis* strain Si3 produces a broad spectrum peoteinaceous antifungal compound. Appl Environ Microbiol. 2001; 67: 1–5. 11133421 10.1128/AEM.67.1.1-5.2001 PMC92504

[23] Lane DJ. 16S/23S rRNA sequencing. In: Stackebrandt E, Goodfellow M, editors. Nucleic acids techniques in bacterial systematic. New York. 1991. p. 115–175.

[24] Turner S, Pryer KM, Miao VPW, Palmer JD. Investigating deep phylogenetic relationships among cyanobacteria and plastids by small subunit of rRNA sequence analysis. J Euk Microbiol. 1999; 46: 327–338. 10461381 10.1111/j.1550-7408.1999.tb04612.x

[25] Salah RB, Trabelsi I, Mansour RB, Lassoued S, Chouayekh H, Bejar S. A new *Lactobacillus plantarum* strain TN8 from the gastrointestinal tract of poultry induces high cytokine production. Anaerobe. 2012; 18: 436–444. 10.1016/j.anaerobe.2012.05.001 22634330

[26] Ehrmann MA, Kurzak P, Bauer J, Vogel RF. Characterization of lactobacilli towards their use as probiotic adjuncts in poultry. J Appl Microbiol. 2002; 92: 966–975. 11972703 10.1046/j.1365-2672.2002.01608.x

[27] Clinical and Laboratory Standards Institute (CLSI). Performance standards for antimicrobial susceptibility testing. Twenty second Informational Supplement. Wayne PA CLSI; 2012.

[28] Maragkoudakis PA, Zoumpopoulou G, Miaris C, Kalantzopoulos G, Pot B, Tsakalidou E. Probiotic potential of *Lactobacillus* strains isolated from dairy products. Int Dairy J. 2006; 16: 189–199.

[29] Lee H, Yoon H, Ji Y, Kim H, Park H, Lee J, et al Functional properties of *Lactobacillus* strains isolated from kimchi. Int J Food Microbiol. 2011; 145: 155–161. 10.1016/j.ijfoodmicro.2010.12.003 21215484

[30] Jakava-Viljanen M, Palva A. Isolation of surface (s) layer protein carrying *Lactobacillus* species from porcine intestine and faeces and characterization of their adhesion properties to different host tissues. Vet Microbiol. 2007; 124: 264–273. 17544232 10.1016/j.vetmic.2007.04.029

[31] Arasu VM, W-Jung M, Ilavenil S, Jane M, Kim DH, Lee KD, et al Isolation and characterization of antifungal compound from *Lactobacillus plantarum* KCC-10 from forage silage with potential beneficial properties. J Appl Microbiol. 2013; 115: 1172–1185. 10.1111/jam.12319 23910250

[32] Chandel U, Pimpalgaonkar R. Efficacy of leaf exudates of *Jatropha curcus* L. on percentage spore germination inhibition of its selected phylloplane and rhizosphere fungi. Indian J Sci Res. 2014; 4: 70–74.

[33] Yang EJ, Chang HC. Purification of a new antifungal compound produced by *Lactobacillus plantarum* AF1 isolated from kimchi. Int J Food Microbiol. 2010; 139: 56–63. 10.1016/j.ijfoodmicro.2010.02.012 20226553

[34] Dalie D, Pinson-Gadias L, Atanasova-Penichon V, Marchegay G, Barreau C, Deschamps A, et al Impact of *Pediococcus pentosaceus* strain L006 and its metabolites on fumonisin biosynthesis by *Fusarium verticillioides*. Food Control. 2012; 23: 405–411.

[35] Sangmanee P, Hongpattarakere T. Inhibitory of multiple antifungal components produced by *Lactobacillus plantarum* K35 on growth, aflatoxin production and ultrastructure alterations of *Aspergillus flavus* and *Aspergillus parasiticus*. Food Control. 2014; 40: 224–233.

[36] Leslie JF, Summerell BA. The *Fusarium* laboratory manual Blackwell Publishing 2006 p. 224–227.

[37] Wang H, yan Y, Wang J, Zhang H, Qi W. Production and characterization of antifungal compounds produced by *Lactobacillus plantarum* IMAU10014. PLoS ONE. 2012; 7: e29452 10.1371/journal.pone.0029452 22276116 PMC3261852

[38] Musikasang H, Tani A, H-kittikun A, Maneerat S. Probiotic potential of lactic acid bacteria isolated from chicken gastrointestinal digestive tract. World J Microb Biot. 2009; 25: 1337–1345.

[39] Park SC, Hwang MH, Kim YH, Kim JC, Song JC, Lee KW, et al Comparison of pH and bile resistance of *Lactobacillus acidophilus* strains isolated from rat, pig, chicken and human sources. World J Microbiol Biotechnol. 2006; 22: 35–37.

[40] Kim PI, Jung MY, Chan YH, Kim S, Kim SJ, Park YH. Probiotic properties of *Lactobacillus Bifidobacterium* strains isolated from porcine gastrointestinal tract. Appl Microbiol Biotechnol. 2007; 74: 1103–1111. 17136367 10.1007/s00253-006-0741-7

[41] Ström K, Schnürer J, Melin P. Co-cultivation of antifungal *Lactobacillus plantarum* MiLAB 393 and *Aspergillus nidulans*, evaluation of effects on fungal growth and protein expression. FEMS Microbiol Lett. 2005; 246: 119–124. 15869970 10.1016/j.femsle.2005.03.047

[42] Gong AD, Li HP, Yuan QS, Song XS, Yao W, He WJ, et al Antagonistic mechanism of Iturin A and Plipastatin A from *Bacillus amyloliquefaciens* S76-3 from wheat spikes against *Fusarium graminearum*. PLoS ONE. 2015; 10: e0116871 10.1371/journal.pone.0116871 25689464 PMC4331432

[43] Mauch A, Bello FD, Coffey A, Arendt EK. The use of *Lactobacillus brevis* PS1 to in vitro inhibit the outgrowth of *Fusarium culmorum* and other common *Fusarium* species found on barley. Int J Food Microbiol. 2010; 141: 116–121. 10.1016/j.ijfoodmicro.2010.05.002 20580986

[44] Koch E, Loffler I. Partial characterization of the antimicrobial activity of *Streptomycesantimycoticus* FZB53. J Phytopathol. 2009; 157: 235–242.

[45] Lahtinen SJ, Haskard CA, Ouwehand AC, Salminen SJ, Ahokas JT. Binding of aflatoxin B1 to cell wall components of *Lactobacillus rhamnosus* strain GG. Food Addit Contam. 2004; 21: 158–164. 14754638 10.1080/02652030310001639521

[46] Niderkorn V, Morgavi DP, Aboab B, Lemaire M, Boudra H. Cell wall component and mycotoxin moieties involved in the binding of fumonisin B1 and B2 by lactic acid bacteria. J Appl Microbiol. 2009; 106: 977–985. 10.1111/j.1365-2672.2008.04065.x 19187153

[47] Pohl CH, Koch LF, Thibaine VS. Antifungal free fatty acids Science against Microbial Pathogens: Communicating current research and technological advance. Mendez-Villas A editor. Formatex 2011.

[48] Masui H, Kondo T, Kojima M. An antifungal compound, 9,12,13-trihydroxy-(E)-10-octadecenoic acid, from *Colocasia antiquorum* inoculated *Ceratocystis fimbriata*. Phytochemistry. 1989; 28: 2613–2615.

[49] Agoramoorthy G, Chandrasekaran M, Venkatesalu V, Hsu MJ. Antibacterial and antifungal activities of fatty acid methyl esters of the blind-your-eye mangrove from India. Braz J Microbiol. 2007: 38: 739–742.

[50] Bhogireddy N, Mathi P, Ambatipudi N, Talluri V, Bokka VR. *In vitro* anti-inflammatory and biofractionation of *Entada pursaetha* DC ethanol seed extract in LPS induced RAW 264.7 macrophage cells. Adv Biol Res. 2015; 9: 109–116.

