# Supplementary figures and images for: Antifungal Attributes of Lactobacillus plantarum MYS6 against Fumonisin Producing Fusarium proliferatum Associated with Poultry Feeds
Source: PLoS One. 2016 Jun 10;11(6):e0155122. doi: 10.1371/journal.pone.0155122 (PMC4902316; doi:10.1371/journal.pone.0155122)

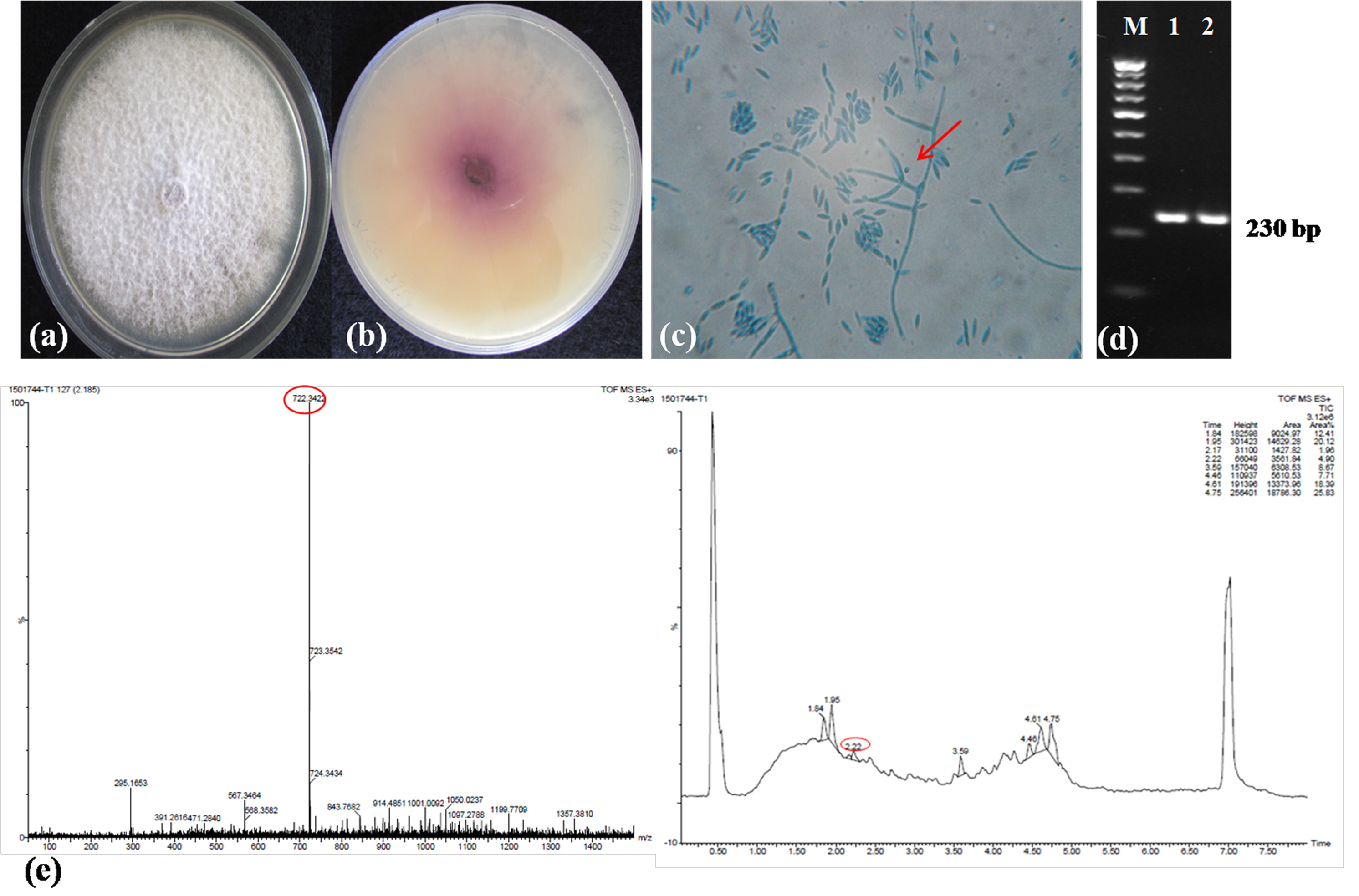

Supplement: S1 Fig — Morphological and molecular identification of F. proliferatum MYS9 (a) Colony morphology,aborse, (b) reverse, (c) micromorphological features showing polyphialides and short conidial chains, (d) Agarose gel showing species specific amplicon size of ~230 bp of F. proliferatum MYS9; M- 100bp DNA ladder, lane 1 –MTCC standard F. proliferatum strain 286, lane 2—F. proliferatum MYS9 (e) Mass spectrometric and liquid chromatogram results confirming fumonisin production by F. proliferatum MYS9 at a retention time 2.18 min having a molar mass of 722 g/mol. (TIFF) [file pone.0155122.s001.tiff]

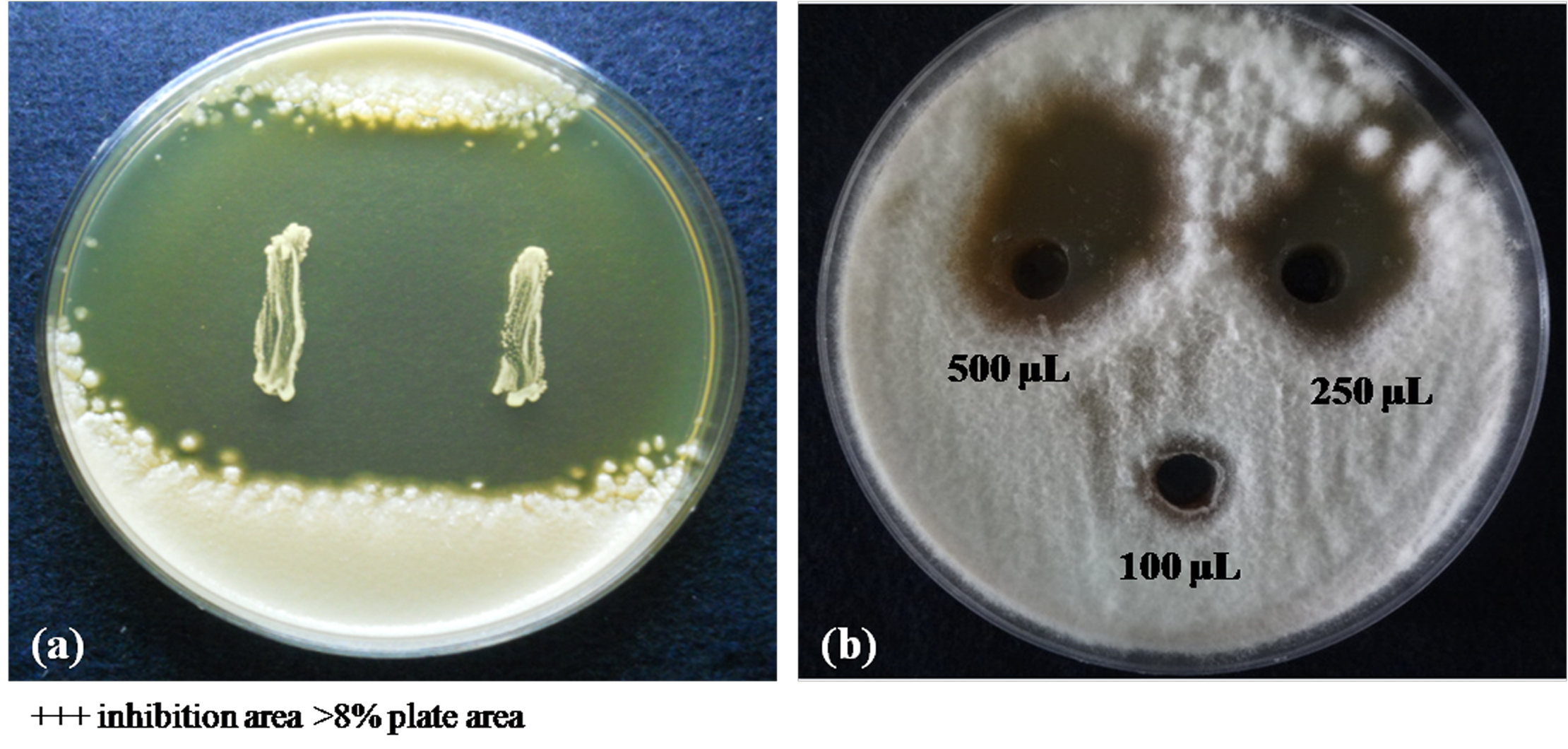

Supplement: S2 Fig — (a) Agar overlay method; a clear inhibition zone is displayed around the two streaks of L. plantarum MYS6 thus showing growth inhibition of F. proliferatumMYS9, (b) Well diffusion method; an evident inhibition of fungal growth increasing with concentration of CFS of L. plantarum MYS6. (TIF) [file pone.0155122.s002.tif]

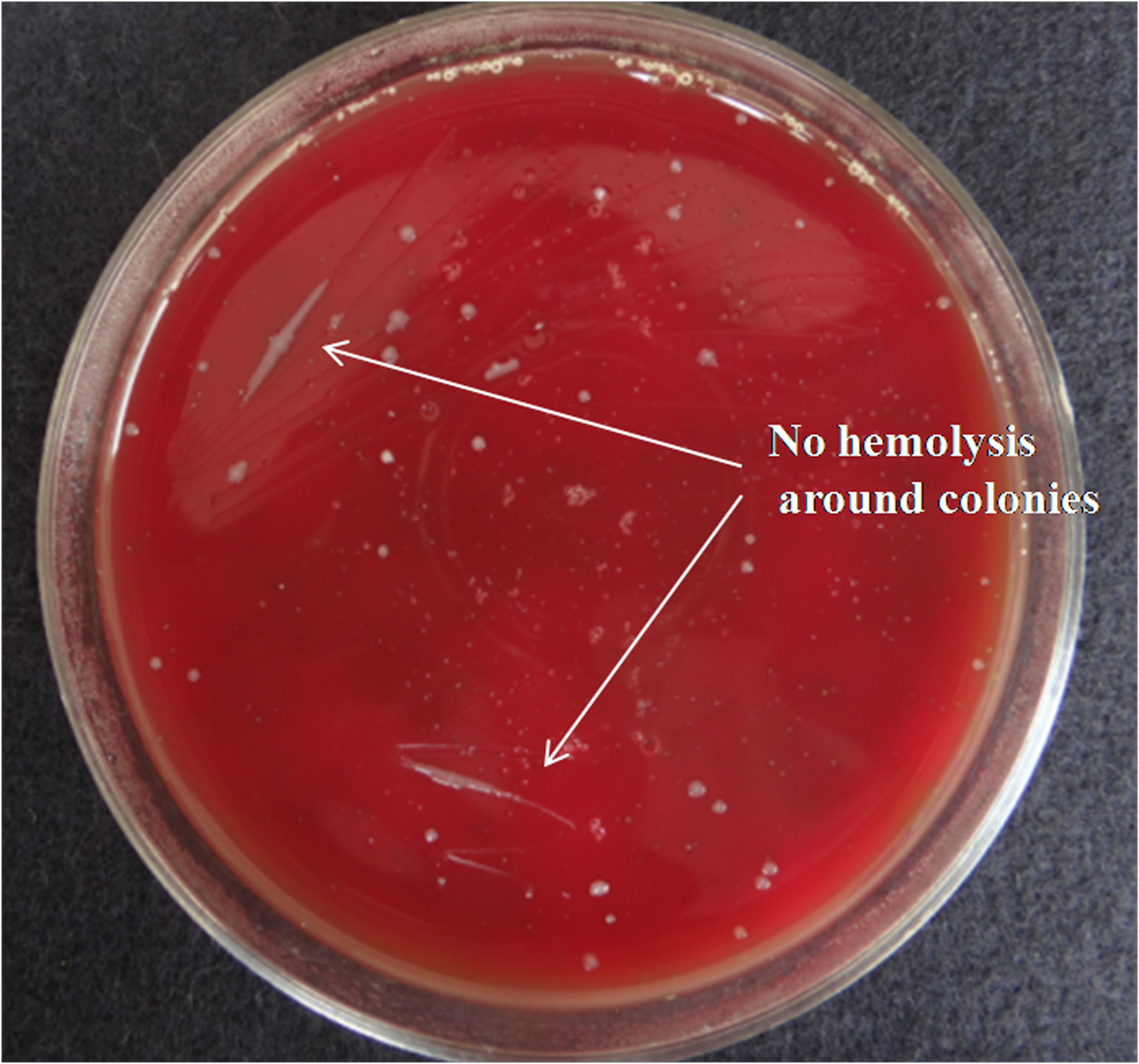

Supplement: S3 Fig — (TIF) [file pone.0155122.s003.tif]

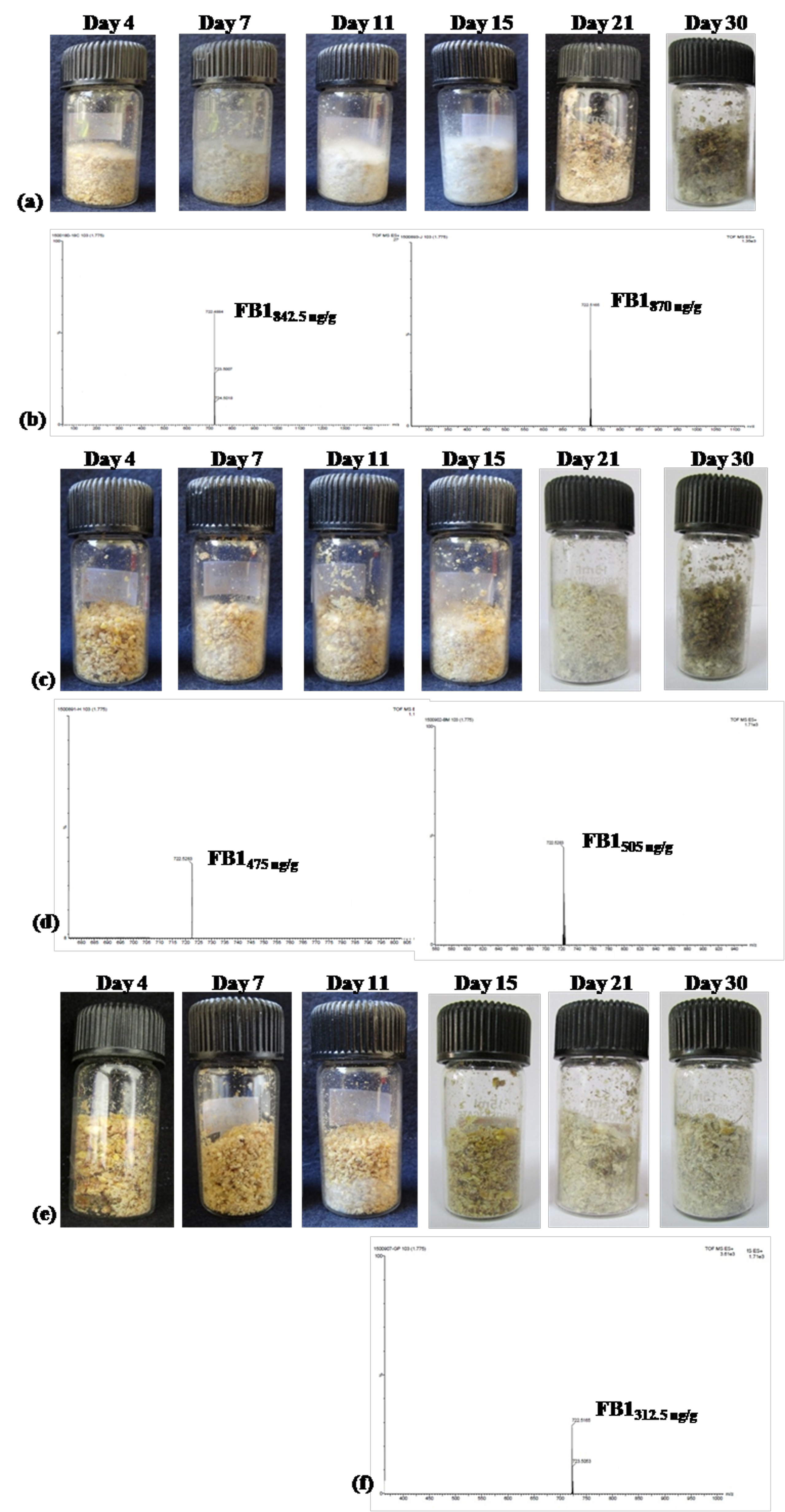

Supplement: S4 Fig — Fumonisin biosynthesis inhibition in poultry feed model (a) control, F. proliferatum MYS9 growth up to 30 days (b) control-mass spectrum of FB1 at 21 and 30 days respectively (c) effect of L. plantarum MYS6 on F. proliferatum MYS9 growth up to 30 days (d) L. plantarum MYS6 treated -mass spectrum of FB1 at 21 and 30 days respectively (e) effect of CFS of L. plantarum MYS6 on F. proliferatum MYS9 growth up to 30 days (f) CFS of L. plantarum MYS6 treated—mass spectrum of FB1 at 21 and 30 days respectively. (TIF) [file pone.0155122.s004.tif]

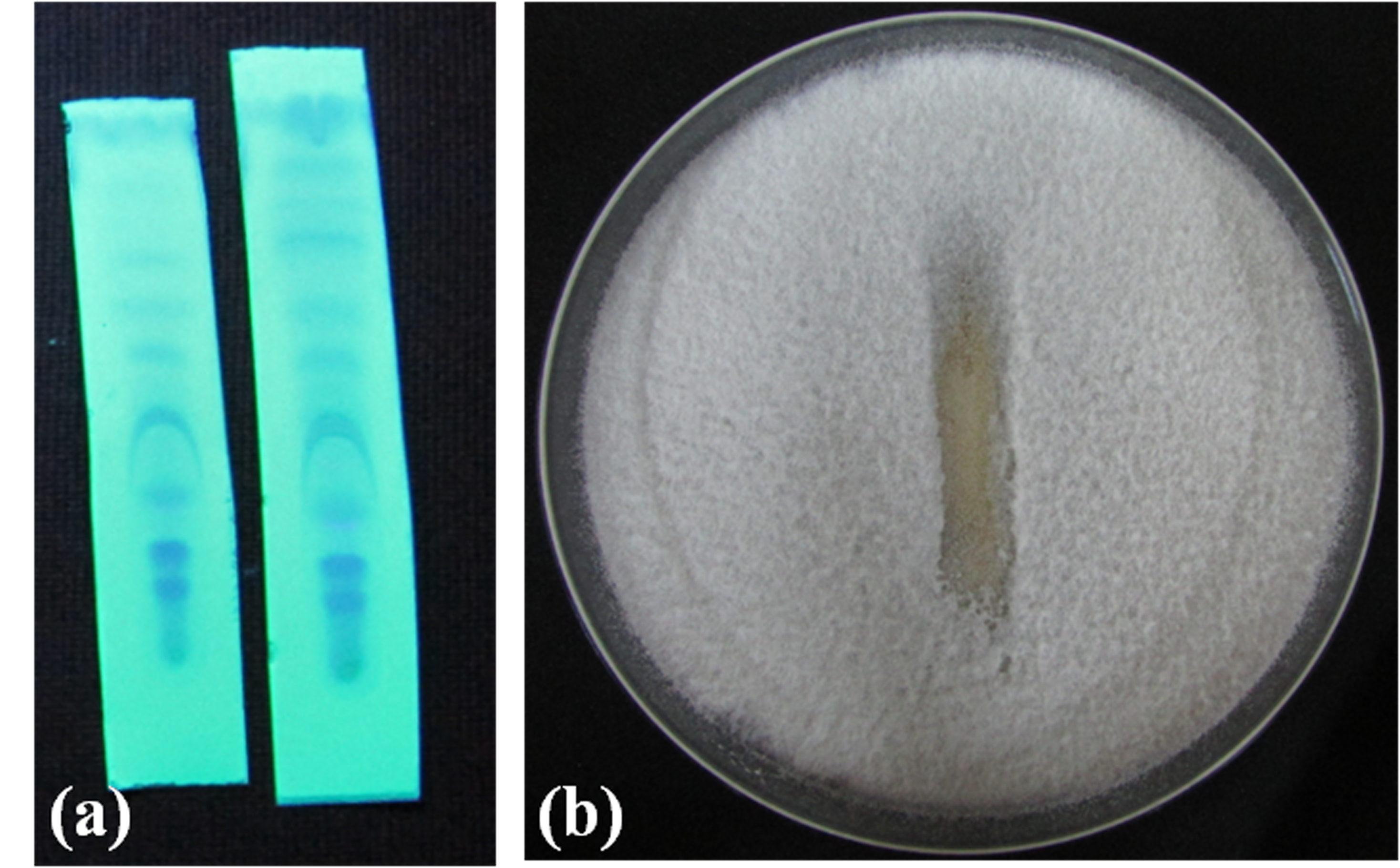

Supplement: S5 Fig — TLC and its Bioautography of CFS of L. plantarum MYS6 (a) TLC separation of CFS in Chloroform:methanol solvent system showing three bands (b) bioautography showing significant inhibition of F. proliferatumMYS9 by TLC purified chloroform:methanol fraction of CFS of L. plantarum MYS6. (TIF) [file pone.0155122.s005.tif]

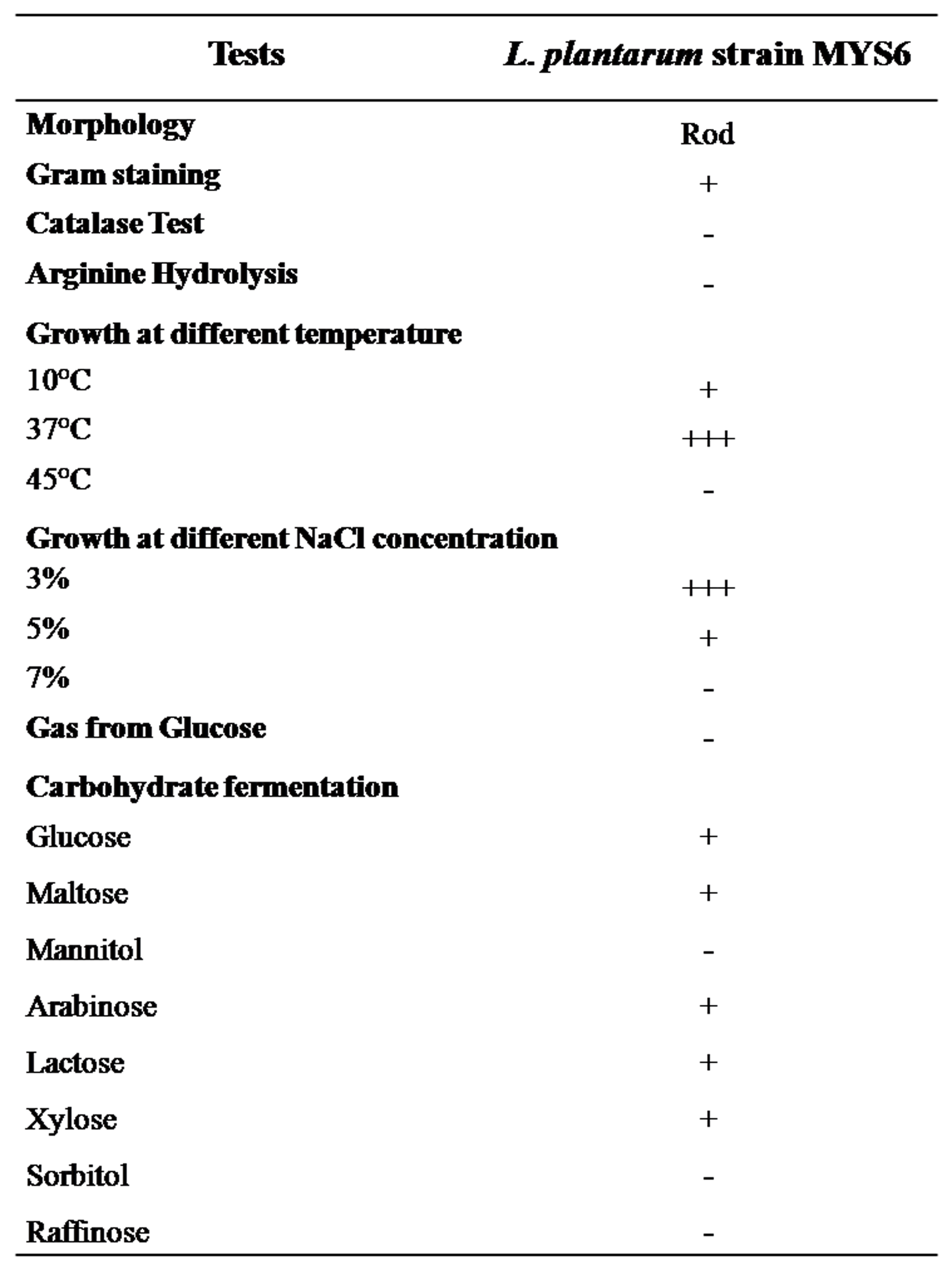

Supplement: S1 Table — (TIF) [file pone.0155122.s007.tif]
