# Supplementary material for: Antifungal Attributes of Lactobacillus plantarum MYS6 against Fumonisin Producing Fusarium proliferatum Associated with Poultry Feeds
Source: PLoS One. 2016 Jun 10;11(6):e0155122. doi: 10.1371/journal.pone.0155122 (PMC4902316; doi:10.1371/journal.pone.0155122)
Supplement: S6 Fig — (DOCX) [file pone.0155122.s006.docx]

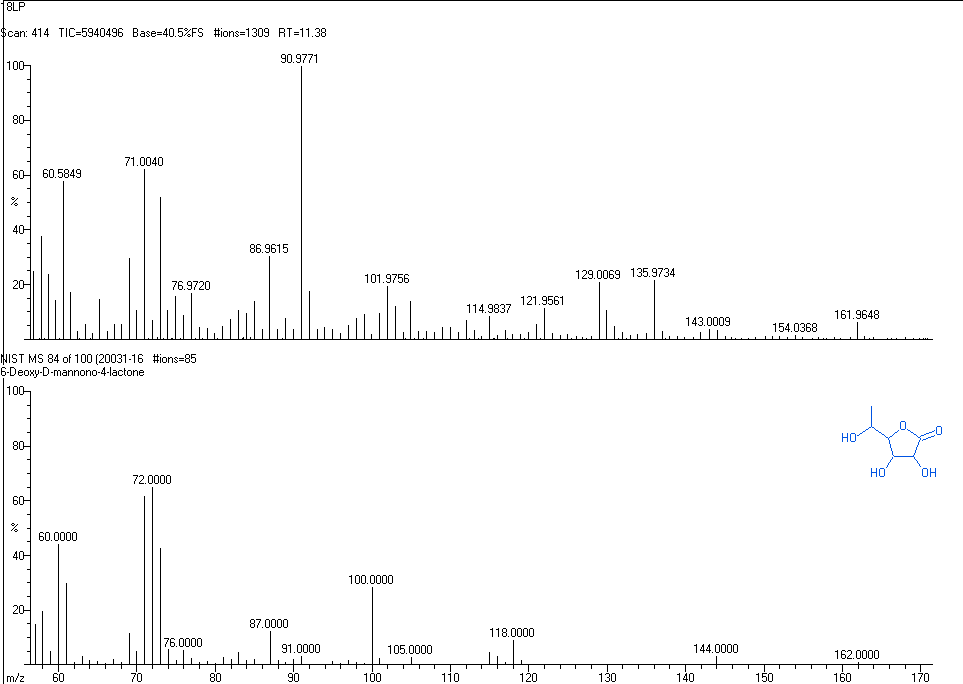


**S6a Fig. MS profile of 6-Deoxy-D-mannono-4-lactone produced by *L. plantarum* MYS6.**


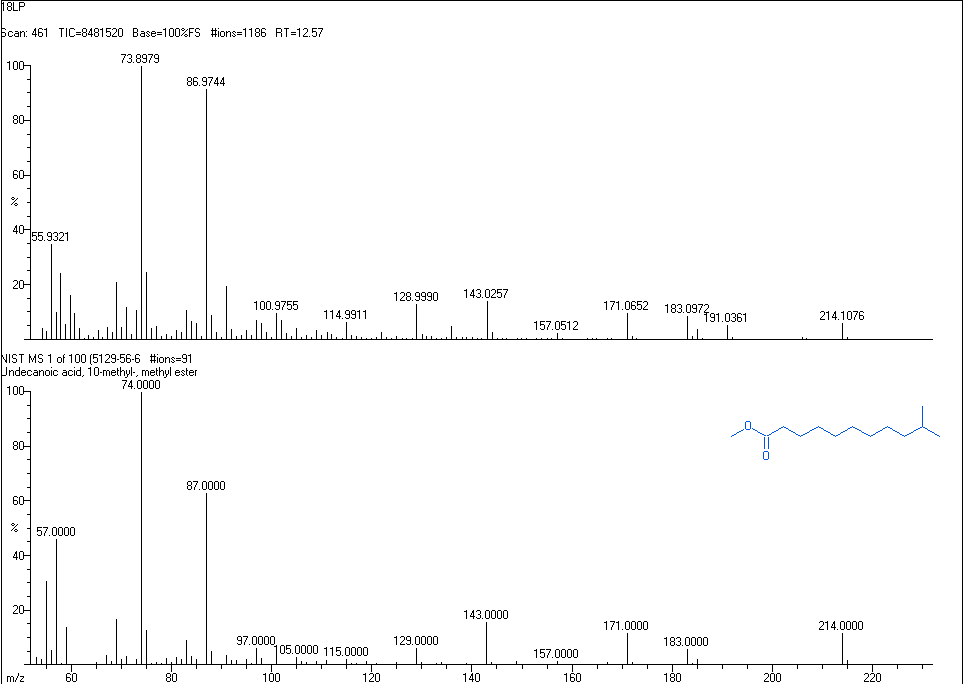


**S6b Fig. MS profile of Undecanoic acid, 10-methyl, methyl ester produced by *L. plantarum* MYS6.**


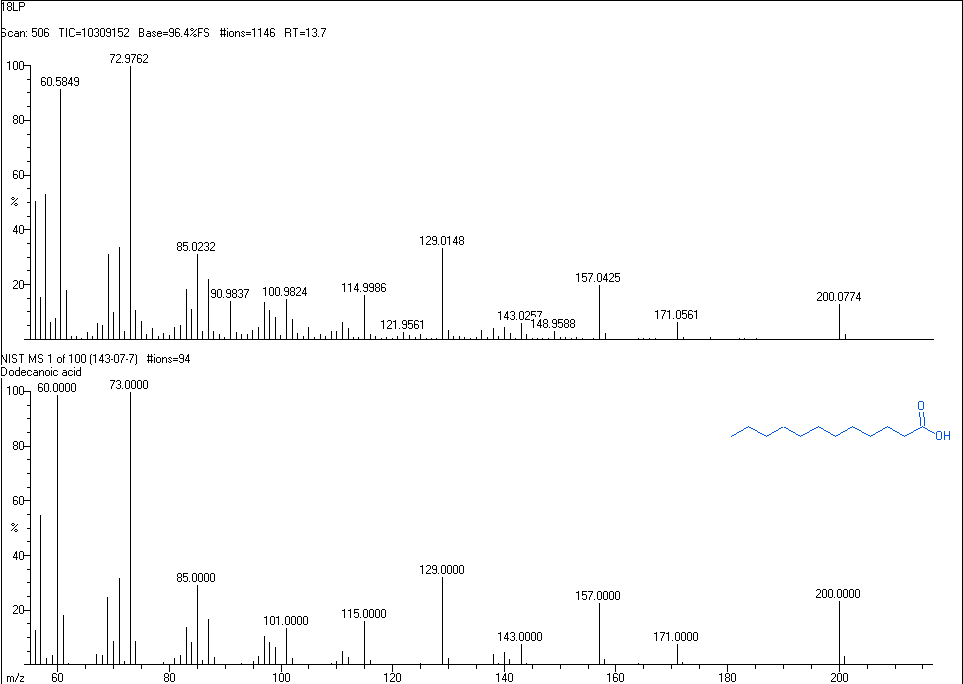


**S6c Fig. MS profile of Dodecanoic acid produced by *L. plantarum* MYS6.**

**
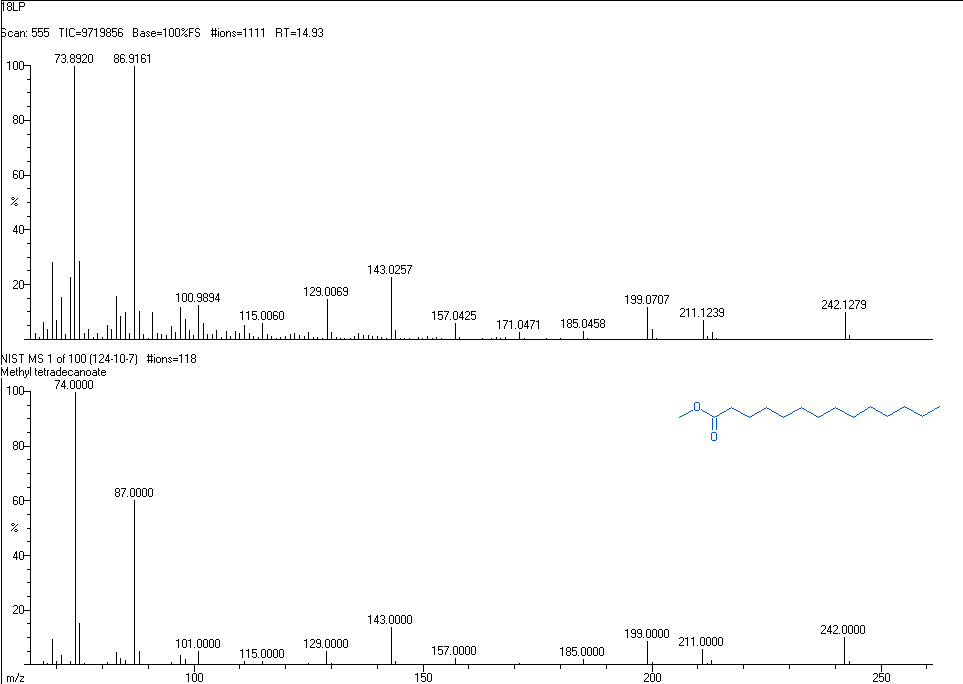
**

**S6d Fig. MS profile of Methyl tetradecanoate produced by *L. plantarum* MYS6.**


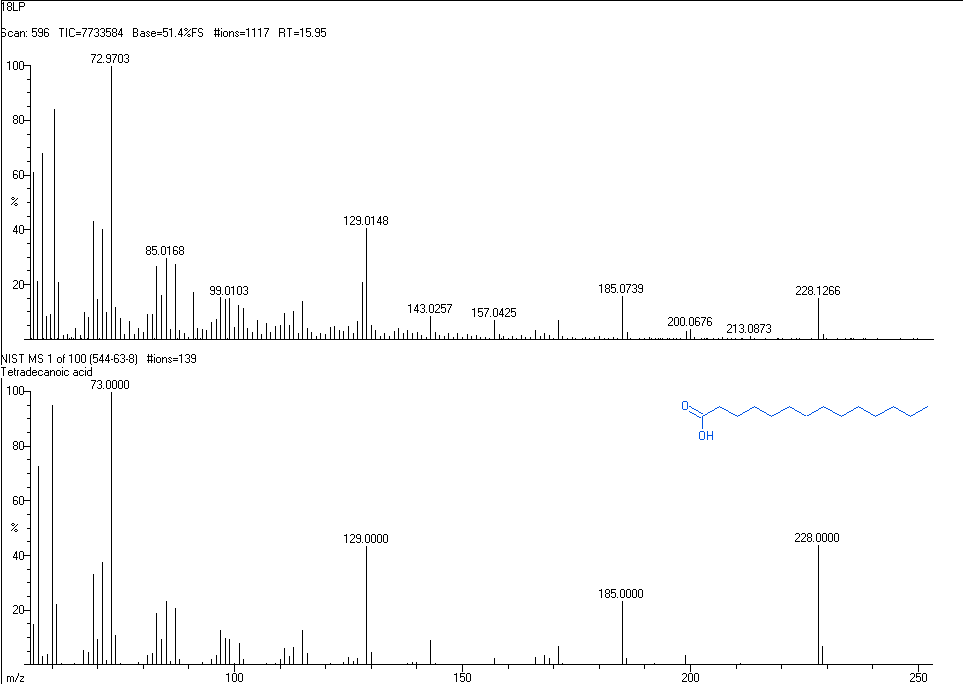


**S6e Fig. MS profile of Tetradecanoic acid produced by *L. plantarum* MYS6.**


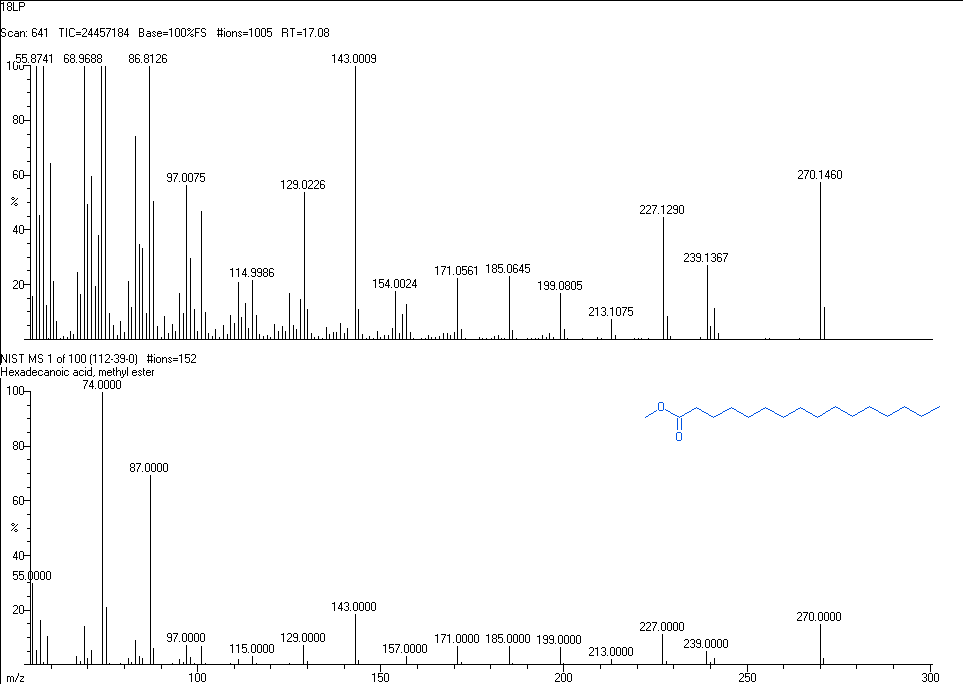


**S6f Fig. MS profile of Hexadecanoic acid, methyl ester produced by *L. plantarum* MYS6.**


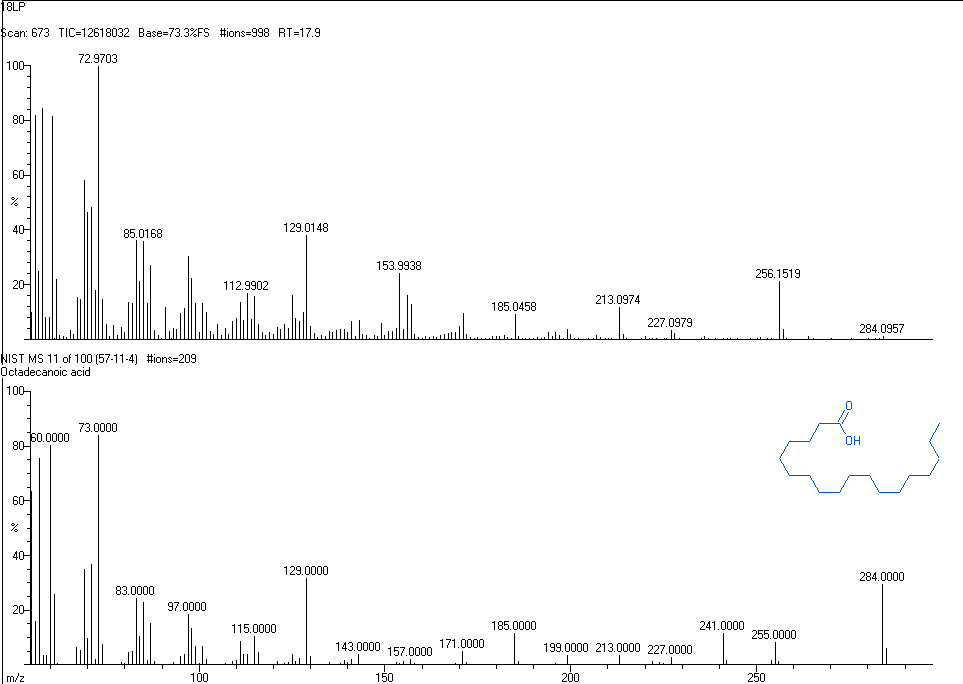


**S6g Fig. MS profile of Octadecanoic acid produced by *L. plantarum* MYS6.**


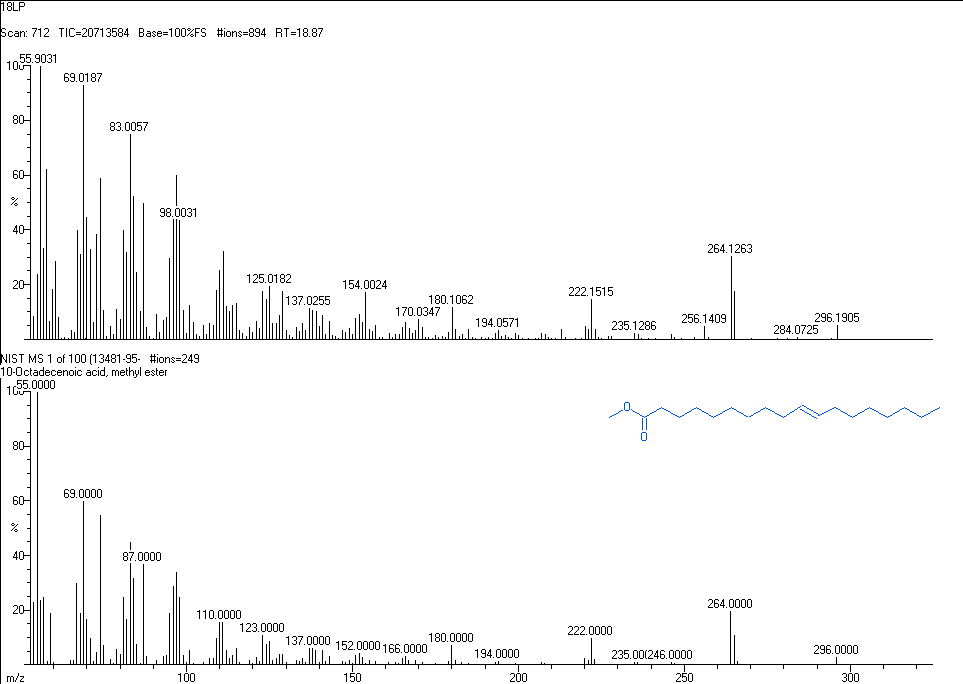


**S6h Fig. MS profile of 10-Octadecenoic acid, methyl ester produced by *L. plantarum* MYS6.**


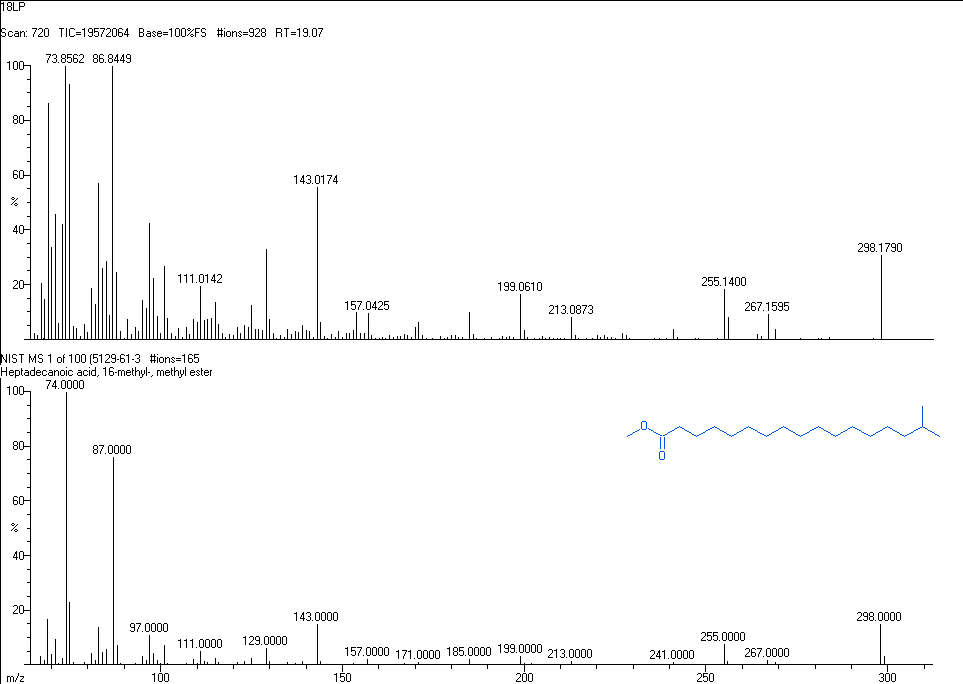


**S6i Fig. MS profile of Heptadecanoic acid, 16-methyl, methyl ester produced by *L. plantarum* MYS6.**


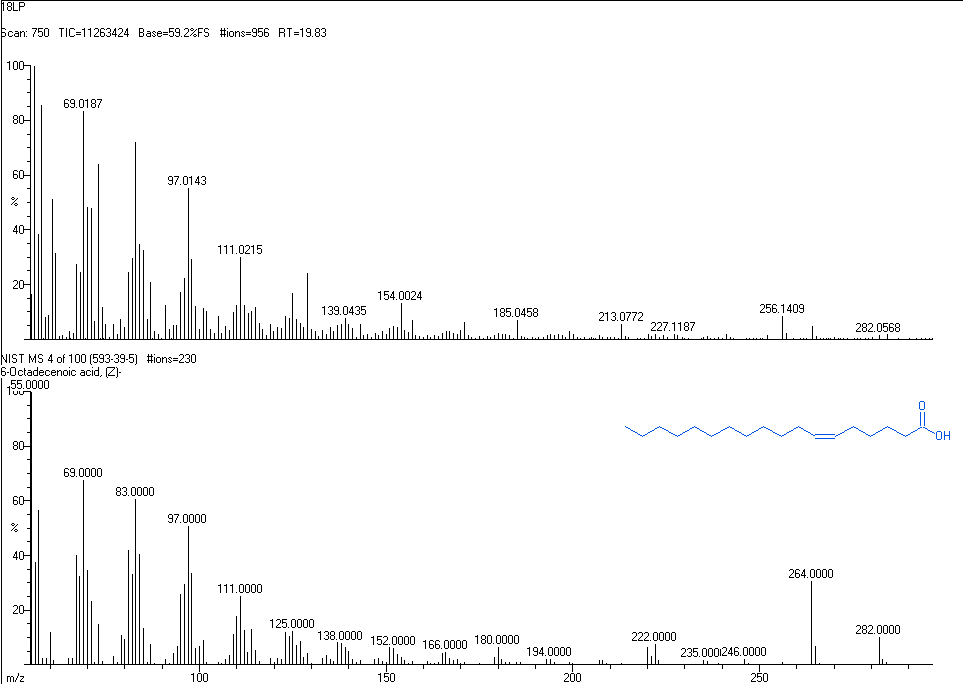


**S6j Fig. MS profile of 6-Octadecenoic acid, [Z] produced by *L. plantarum* MYS6.**


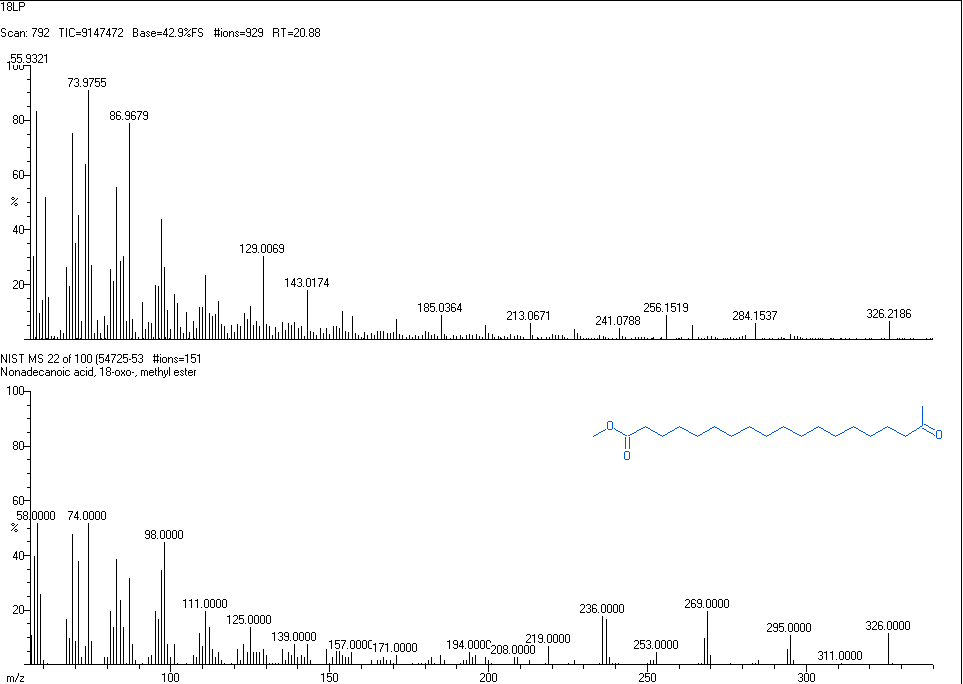


**S6k Fig. MS profile of Nonadecanoic acid, 18-oxo, methyl ester produced by *L. plantarum* MYS6.**


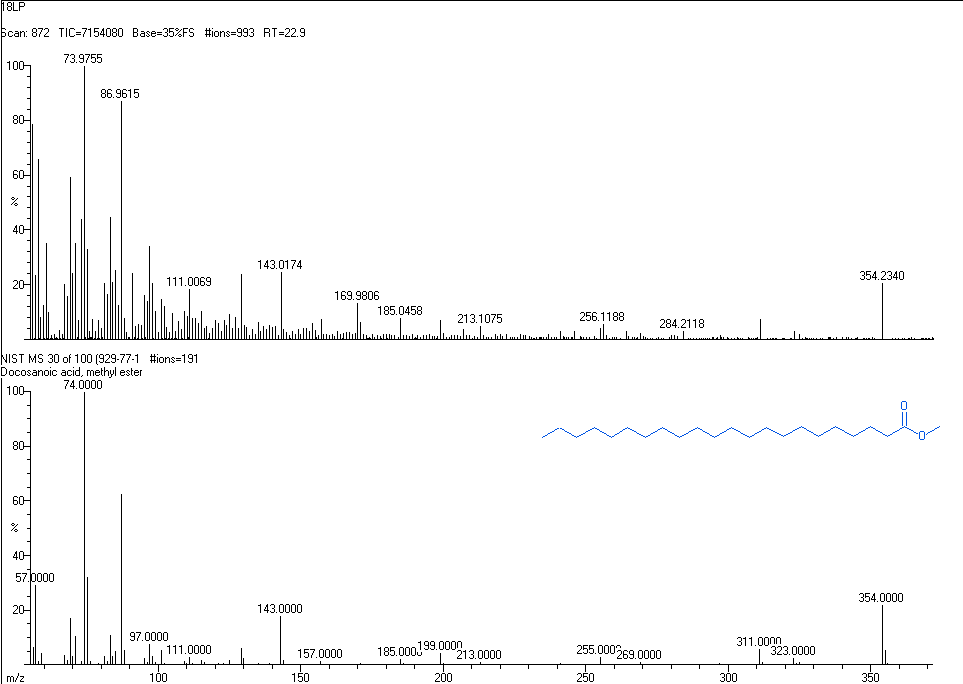


**S6l Fig. MS profile of Docosanoic acid, methyl ester produced by *L. plantarum* MYS6.**
